# Tumor-specific cholinergic CD4^+^ T lymphocytes guide immunosurveillance of hepatocellular carcinoma

**DOI:** 10.1038/s43018-023-00624-w

**Published:** 2023-08-28

**Authors:** Chunxing Zheng, Bryan E. Snow, Andrew J. Elia, Robert Nechanitzky, Carmen Dominguez-Brauer, Shaofeng Liu, Yin Tong, Maureen A. Cox, Enrico Focaccia, Andrew C. Wakeham, Jillian Haight, Chantal Tobin, Kelsey Hodgson, Kyle T. Gill, Wei Ma, Thorsten Berger, Mathias Heikenwälder, Mary E. Saunders, Jerome Fortin, Suet Yi Leung, Tak W. Mak

**Affiliations:** 1 Princess Margaret Cancer Centre, University Health Network, Toronto, Ontario Canada; 2 Centre for Oncology and Immunology, Hong Kong Science Park, Hong Kong SAR, China; 3 Tumor Immunotherapy Program, Princess Margaret Cancer Centre, University Health Network, Toronto, Ontario Canada; 4 Department of Pathology, School of Clinical Medicine, Li Ka Shing Faculty of Medicine, The University of Hong Kong, Queen Mary Hospital, Pokfulam, Hong Kong SAR, China; 5 Division of Chronic Inflammation and Cancer, German Cancer Research Center (DKFZ), Heidelberg, Germany; 6 The M3 Research Center, Medical Faculty Tübingen, Tübingen, Germany; 7 Departments of Immunology and Medical Biophysics, University of Toronto, Toronto, Ontario Canada; 8 Present Address: Department of Microbiology and Immunology, University of Oklahoma Health Sciences Center, Oklahoma City, OK USA

**Keywords:** Immunosurveillance, Hepatocellular carcinoma, Tumour immunology, Cancer

## Abstract

Cholinergic nerves are involved in tumor progression and dissemination. In contrast to other visceral tissues, cholinergic innervation in the hepatic parenchyma is poorly detected. It remains unclear whether there is any form of cholinergic regulation of liver cancer. Here, we show that cholinergic T cells curtail the development of liver cancer by supporting antitumor immune responses. In a mouse multihit model of hepatocellular carcinoma (HCC), we observed activation of the adaptive immune response and induction of two populations of CD4^+^ T cells expressing choline acetyltransferase (ChAT), including regulatory T cells and dysfunctional PD-1^+^ T cells. Tumor antigens drove the clonal expansion of these cholinergic T cells in HCC. Genetic ablation of *Chat* in T cells led to an increased prevalence of preneoplastic cells and exacerbated liver cancer due to compromised antitumor immunity. Mechanistically, the cholinergic activity intrinsic in T cells constrained Ca^2+^–NFAT signaling induced by T cell antigen receptor engagement. Without this cholinergic modulation, hyperactivated CD25^+^ T regulatory cells and dysregulated PD-1^+^ T cells impaired HCC immunosurveillance. Our results unveil a previously unappreciated role for cholinergic T cells in liver cancer immunobiology.

## Abstract (web summary)

Zheng et al. show that ChAT-expressing cholinergic T cells suppress the development of liver cancer via their cholinergic activity, which constrains calcium/NFAT signaling induced by TCR engagement.

## Main

Hepatocellular carcinoma (HCC) is the most common primary liver malignancy in humans and the third most common cause of cancer-related deaths worldwide^1^. Risk factors for HCC include infection with hepatitis B virus or hepatitis C virus, excessive alcohol consumption, non-alcoholic fatty liver disease and aflatoxins^2^. These extrinsic risk factors cause chronic hepatitis that cooperates with intrinsic factors, particularly the accumulation of genetic mutations, to set the stage for HCC development^3,4^.

The immune system plays a dual role in liver cancer and can sense and eliminate preneoplastic and malignant hepatocytes^5,6^; it can also promote the selection of tumor cells and favor cancer progression in situations of chronic inflammation or immunosuppression^7–9^. This duality renders current immunotherapies that target immune checkpoints suboptimal for HCC^8^. Further exploration of the molecular determinants of immune responses in liver cancer is necessary to understand HCC biology and guide the design of effective therapeutic strategies.

The nervous system is involved in the development of cancer in multiple tissues^10–12^. For example, cholinergic fibers infiltrate prostate tumors, promoting their invasion and metastasis^11^. Engagement of nicotinic acetylcholine (ACh) receptors (nAChRs) mediates lung cancer growth^13^. Vagal innervation promotes gastric tumorigenesis through the M3 muscarinic ACh receptor (mAChR)^10^. Extensive efforts have been directed at delineating the hepatic nervous system. Despite some discrepancies among studies, sympathetic and parasympathetic neural markers have been detected in regions of the hepatic artery, portal vein and bile ducts in the majority of species investigated^14^. However, the liver parenchyma of rodents and humans appears to be devoid of vagal or cholinergic innervation, as determined by immunohistochemistry, retrograde tracing and advanced three-dimensional imaging^15–17^. Thus, whether and how cholinergic signaling plays a role in HCC regulation remains an open question.

In this study, we establish that subpopulations of CD4^+^ T cells expressing choline acetyltransferase (ChAT), the rate-limiting enzyme governing ACh synthesis, are induced during the development of liver cancer in mice. Importantly, we show that genetic ablation of *Chat* in T cells impairs HCC immunosurveillance. Examination of data from human HCC samples revealed parallels to our mouse findings. Our results demonstrate an unexpected aspect of the regulation of cancer immunosurveillance: mediation by an immune cell-derived neurotransmitter.

## Results

### Induction of HCC using CRISPR and transposon technology

**Fig. 1:**
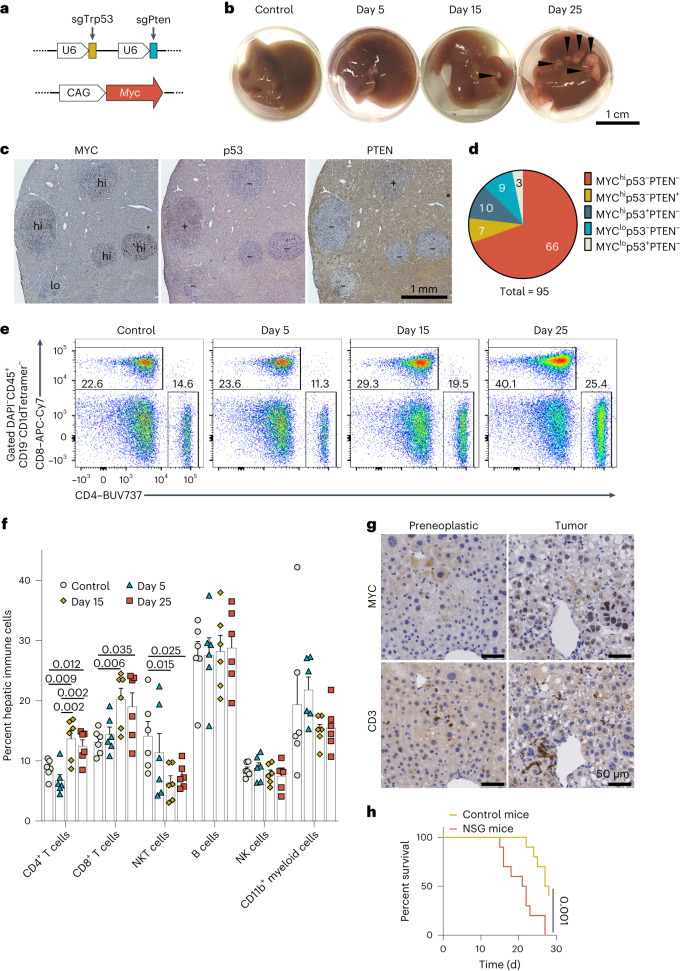
Immunosurveillance is present in CRISPR- and transposon-induced HCC in mice. **a**, Schematic diagrams of the plasmids used to induce CRISPR–Cas9-mediated deletion of *Trp53* and *Pten* (top) and transposon-mediated overexpression of *Myc* in mouse livers (bottom). **b**, Representative macroscopic views of livers from mice injected with the combination of plasmids shown in **a** at the indicated days after injection. Control mice received transposase vector only. Arrowheads indicate tumor nodules. **c**, Representative histological sections of tumor-burdened livers immunostained to detect MYC, p53 and PTEN. Images are representative of two independent experiments. **d**, Distribution of immunostained liver tumor nodules from the livers in **c**. Numbers of tumor nodules with the indicated immunostaining patterns are labeled in the pie plot, which is a summary of two independent experiments. Sections were resected from three mice per group on day 25 of HCC induction. **e**,**f**, Representative flow cytometry plots (**e**) and quantification (**f**) showing changes in the percentages of the indicated immune cell populations during the development of liver cancer in mice. In **f**, each dot represents an individual mouse (*n* = 6 mice per condition). Data are shown as mean ± s.e.m. Significance was assessed by unpaired, two-tailed *t*-test, and data are representative of three independent experiments. Control mice received transposase vector only. **g**, Representative histological sections from HCC-bearing livers that were immunostained to detect MYC or CD3 in areas of either preneoplastic cells (left) or HCC cells (right). Images represent immunostaining of liver sections from ten mice in one experiment. **h**, Survival of immunodeficient (NSG) and control wild-type (WT) mice (*n* = 10 per group) following injection of *Trp53*/*Pten* CRISPR and *Myc* overexpression plasmids to induce HCC development. *P* = 0.001 by log-rank test. Source data

We sought to model HCC in mice by combining genetic alterations recurrently observed in human disease. These changes included mutation of the *TP53* and *PTEN* tumor suppressor genes and overexpression of the *MYC* oncogene^18–21^. CRISPR-mediated somatic knockout of tumor suppressor genes and transposon-based expression of oncogenes induce HCC in mice^6,22,23^. To mimic the multihit process of human liver carcinogenesis, we combined these two approaches by ablating *Trp53* and *Pten* using duplex CRISPR and overexpressing *Myc* using the Sleeping Beauty transposon (Fig. 1a). These vectors were delivered in combination to mice via hydrodynamic injection, allowing for specific plasmid delivery to hepatocytes. Synergism between *Myc* expression and *Trp53*/*Pten* ablation induced rapid HCC development. Neoplasms were visible on the liver surface by 15 d after injection, with substantial tumor nodules present by day 25 (Fig. 1b). Immunostaining confirmed that the majority of these tumor clones were negative for p53 and PTEN and positive for MYC (Fig. 1c,d).

**Extended Data Fig. 1:**
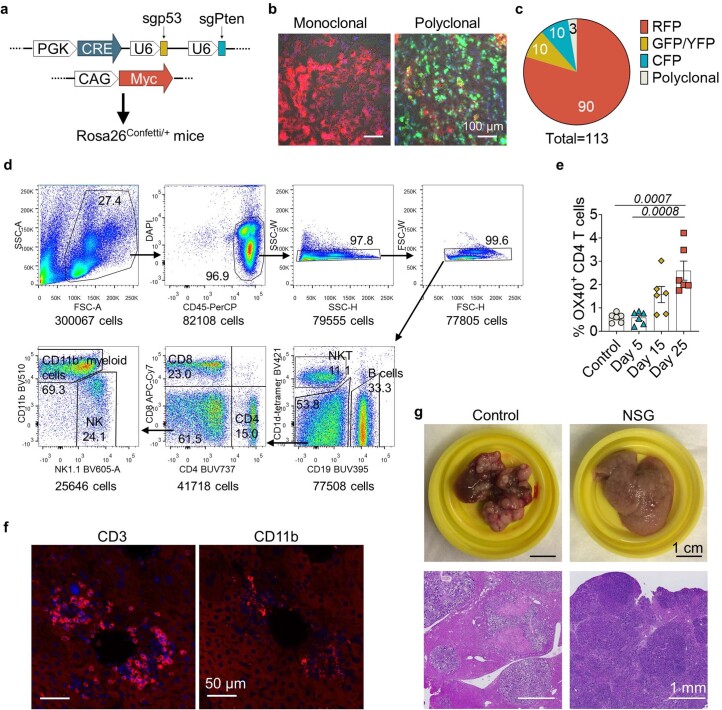
The immunosurveillance of murine liver cancer. **a** Schematic diagrams of the plasmids used to simultaneously induce CRISPR/Cas9-mediated deletion of *Trp53* and *Pten*, *Cre* expression, and *Myc* overexpression in *Rosa26*^*Confetti*/+^ reporter mice. **b** Representative fluorescence microscopy images depicting (left) a monoclonal and (right) a polyclonal tumor from the livers of the mice in (a). Clonality was determined based on the expression of one or multiple fluorescent markers encoded by the *Rosa26*^*Confetti*^ reporter, representative of one experiment. **c** Quantification of monoclonal and polyclonal tumors expressing the indicated fluorescent markers in livers from the mice in (a). Numbers of tumor nodules expressing the indicated fluorescent marker(s) are labeled in the pie plot. Data are from examination of 113 tumor nodules in liver sections from 10 *Rosa26*^*Confetti*^ reporter mice. **d** Gating strategy used for flow cytometric analysis of murine hepatic immune cells. **e** Percentage of OX40^+^ CD4^+^ T cells in livers on the indicated days of liver cancer development. Each dot represents an individual mouse (n=6 mice per group). Data are the mean ± SEM. P values were determined by unpaired, two-tailed t-test. **f** Representative immunofluorescence images showing CD3 and CD11b expression in adjacent sections of an HCC-bearing liver, representative of one experiment. **g** Representative macroscopic images (upper) and microscopic images (lower) of tumor-bearing livers resected at the humane endpoint from NSG and control mice subjected to HCC induction, representative of one experiment. Related to Fig. 1. Source data

We engineered the CRISPR vector to also express Cre recombinase (Extended Data Fig. 1a). The transposon and Cre*-*encoding CRISPR vectors were delivered into *Rosa26*^Confetti/+^ mice, animals in which cells stochastically express one of four fluorescent proteins after Cre-mediated recombination^24^. We found that most tumor nodules expressed a single fluorescent marker, suggesting that these malignancies were monoclonal (Extended Data Fig. 1b,c).

### Immunosurveillance is elicited in our HCC model

The immune microenvironment shapes the clonal evolution of tumor cells^7,25^. We investigated whether immune responses were evoked during tumorigenesis in our model. We found that hepatic CD4^+^ T cells and CD8^+^ T cells expanded as HCC development progressed, whereas the percentage of natural killer T (NKT) cells was reduced (Fig. 1e,f and Extended Data Fig. 1d). Analysis of OX40, a transient marker of T cell antigen receptor (TCR) activation, showed that antigen-stimulated CD4^+^ T cells were increased (Extended Data Fig. 1e). The infiltrating immune cells, including CD3^+^ T cells and CD11b^+^ antigen-presenting cells (APCs), were positioned around MYC^+^ preneoplastic cells and in established HCC (Fig. 1g and Extended Data Fig. 1f). Therefore, immune responses, particularly those mediated by T cells, are activated in our HCC model.

To determine the overall role of the immune system in our model, we induced HCC in severely immunodeficient NOD *scid* gamma (NSG) mice. Compared to immunocompetent animals, NSG mice developed more severe disease and died sooner, with tumor cells diffused throughout the liver instead of confined in discrete nodules (Fig. 1h and Extended Data Fig. 1g). Thus, immune cells participate in protection against liver cancer development in this setting.

### ChAT-expressing T cells are induced during HCC development

**Fig. 2:**
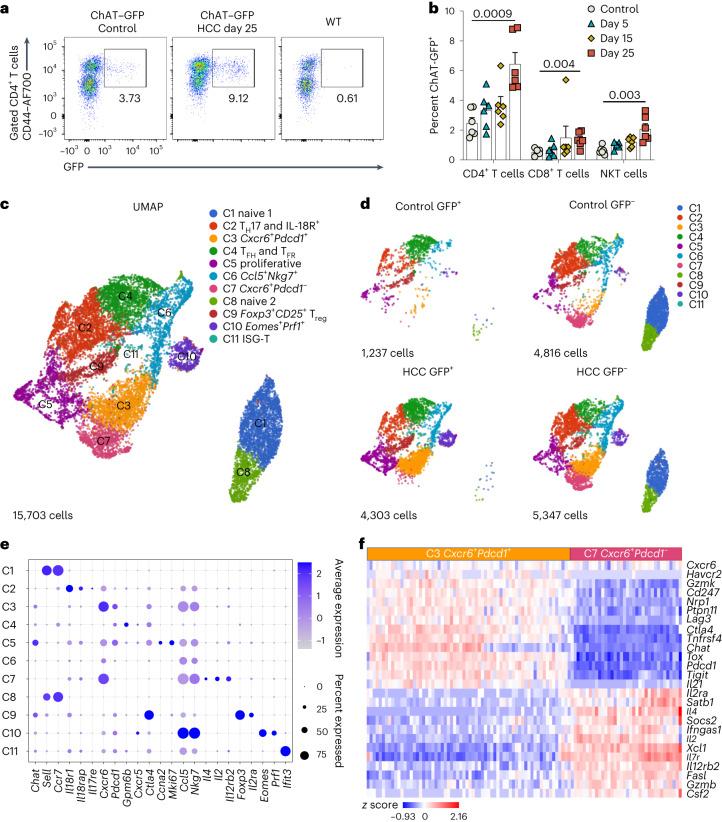
ChAT-expressing T cells are induced during HCC development. **a**,**b**, Representative flow cytometry plots (**a**) and quantification (**b**) of GFP expression in the indicated T cell subsets during HCC progression in *Chat-GFP* reporter mice. In **b**, each dot represents an individual mouse (*n* = 6 mice per condition). Data are shown as mean ± s.e.m. *P* values were determined by unpaired, two-tailed *t*-test, and data are representative of three independent experiments. **c**,**d**, Transcriptional landscape of ChAT–GFP^+^ and ChAT–GFP^−^ CD4^+^ T cells in livers from control mice and mice with HCC. Uniform manifold approximation and projection (UMAP) representation of total hepatic CD4^+^ T cells (a total of 15,703 cells; **c**) and CD4^+^ T cells split according to GFP expression and HCC conditions (**d**) based on scRNA-seq analysis. ChAT–GFP^+^ and ChAT–GFP^−^ CD4^+^ T cells were sorted from four mice with HCC and from four control mice. Cells from each mouse were stained with unique barcoded antibodies; ISG-T, interferon-stimulated gene-expressing T cells. **e**, Bubble plot comparing expression of *Chat* and the indicated marker genes across the 11 clusters in **c** and **d**. **f**, Heat map depicting the relative expression of the indicated genes in cluster C3 (*Cxcr6*^+^*Pdcd1*^+^) and cluster C7 (*Cxcr6*^+^*Pdcd1*^–^). Source data

**Extended Data Fig. 2:**
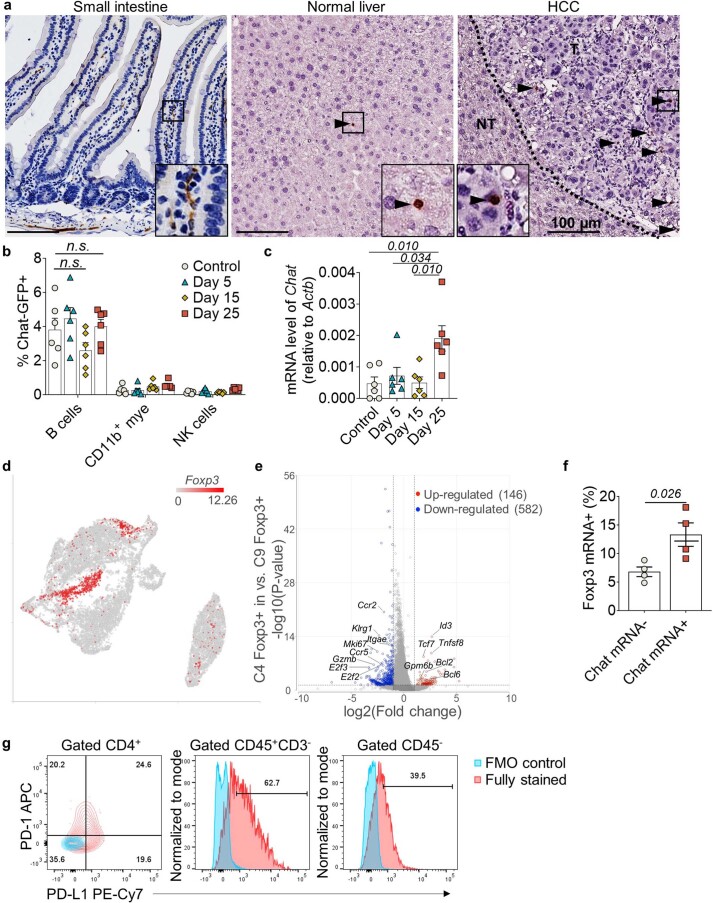
Identification of cholinergic cells in liver cancer. **a** Representative histological sections showing GFP immunoreactivity in (left) small intestine, (middle) normal liver, and (right) HCC-bearing liver resected from *Chat-GFP* mice. Arrowheads, cells with GFP immunoreactivity. Inset boxes show higher magnification views of the GFP^+^ cells in the small boxes. Scale bars, 100 μm. T, tumor tissue. NT, non-tumor tissue. The micrographs displayed are representative of sections obtained from three independent experiments. **b** Percentage of GFP^+^ cells among the indicated cell subsets in livers of *Chat-GFP* mice at the indicated time points following HCC induction. Each dot represents an individual mouse (n=6 mice per group). Data are the mean ± SEM. *n.s*., not significant by unpaired, two-tailed t-test was performed. Results are representative of three independent experiments. **c** qPCR determinations of *Chat* mRNA levels (relative to *Actb*) in hepatic mononuclear cells of *Chat-GFP* mice at the indicated time points following standard HCC induction. Each dot represents an individual mouse. P values were determined by unpaired, two-tailed t-test. **d** Expression of *Foxp3* in CD4^+^ T cells from control and HCC-bearing livers as determined in the scRNAseq UMAP plot shown in Fig. 2c. **e** Volcano plot comparing transcripts between *Foxp3* mRNA-expressing cells from cluster 4 and those from cluster 9. Gene-set analysis (GSA) was performed to identify sets of differentially expressed genes. **f** Percentages of *Foxp3* mRNA^+^ cells among *Chat* mRNA^+^ CD4^+^ T cells and *Chat* mRNA^-^ CD4^+^ T cells from HCC-bearing livers as determined from scRNAseq data. Cells from each mouse were identified using antibody barcodes. Each dot represents an individual mouse (n=4 mice per group). Data are the mean ± SEM. P values were determined by unpaired, two-tailed t-test. **g** Representative flow cytometry plots showing the expression of PD-L1 by CD4^+^ T cells, CD45^+^CD3^-^ immune cells and CD45^-^ cells from HCC-bearing liver. Related to Fig. 2. Source data

Our group has been studying the function of cholinergic T cells in various contexts^26–28^. We analyzed the expression pattern of ChAT in liver tissues of reporter mice expressing green fluorescent protein (GFP) under the control of transcriptional regulatory elements for ChAT (*Chat-GFP* reporter mice). In contrast to the extensive cholinergic neural fibers and plexuses in the small intestine, we found no ChAT-expressing neural fibers in either the parenchyma of normal liver or in HCC (Extended Data Fig. 2a), findings in line with earlier reports^15–17^. However, we observed accumulation of lymphocyte-like ChAT-expressing cells in HCC (Extended Data Fig. 2a). These ChAT–GFP^+^CD4^+^ T cells were comprised mainly of CD44^+^ activated T cells that significantly increased during HCC progression (Fig. 2a,b). The percentages of ChAT–GFP^+^CD8^+^ T cells and NKT cells were also significantly elevated in HCC but to a lesser extent (Fig. 2b). In comparison, the percentage of ChAT–GFP^+^ B cells did not differ between control and HCC-bearing livers, and the expression of ChAT–GFP by NK cells and CD11b^+^ myeloid cells was negligible (Extended Data Fig. 2b). The overall level of *Chat* mRNA in bulk intrahepatic mononuclear cells (MNCs) from HCC-bearing livers was enhanced, reflecting the induction of ChAT-expressing T cells (Extended Data Fig. 2c). Therefore, lymphocytes are the dominant cholinergic cells in both healthy and HCC-bearing livers, and ChAT-expressing T cells are induced during tumorigenesis.

### Transcriptional landscape of cholinergic CD4^+^ T cells in HCC

To delineate the heterogeneity of cholinergic CD4^+^ T cells in HCC, we conducted single-cell RNA sequencing (scRNA-seq) on sorted ChAT–GFP^+^ and ChAT–GFP^−^ CD4^+^ T cells from four control and four HCC-bearing livers. Cells from individual mice were labeled with distinct antibody barcodes, pooled and processed for CITE-seq coupled with TCR-seq. In total, 11 clusters of CD4^+^ T cells were identified: clusters C1 and C8 were two naive T cell clusters; C2 was composed of T helper 17 (T_H_17) cells and cells expressing IL-18 receptor genes; C3 cells coexpressed *Cxcr6* and *Pdcd1*; C4 cells contained follicular helper T (T_FH_) cells and follicular regulatory T (T_FR_) cells; C5 cells were actively cycling; C6 cells expressed *Ccl5* and *Nkg7* but few other markers; C7 cells expressed *Cxcr6* but were negative for *Pdcd1*; C9 cells were canonical regulatory T (T_reg_) cells; C10 cells expressed *Eomes*, *Prf1*, *Gzmk*, *Fasl*, *Gzmb* and other cytotoxic genes; and C11 was a minor cluster expressing interferon (IFN)-stimulated genes (Fig. 2c–e). We observed a marked shift from naive T cells to effector T cells in the presence of HCC and, in particular, the induction of the C3 cluster.

Compared to ChAT–GFP^−^ cells, the ChAT–GFP^+^ population lacked the naive T cell clusters as well as C7, with C2 cells also underrepresented (Fig. 2d). Cells in the C3, C4, C9 and C10 clusters were overrepresented among HCC ChAT–GFP^+^ T cells (Fig. 2d). We identified two subsets of *Foxp3*-expressing T cells, one in C4 and the other in C9. Comparing their transcriptomes, we discerned that the *Foxp3*^+^ cells in C4 were T_FR_ cells that overexpressed *Bcl6*, *Tcf7*, *Gpm6b* and other T_FR_-associated genes and underexpressed *Itgae* and *Gzmb* (Extended Data Fig. 2d,e); this signature is consistent with previous reports^29,30^. Across all four HCC-bearing mice, *Foxp3*^+^ cells were significantly enriched among ChAT-expressing T cells (Extended Data Fig. 2f).

*Cxcr6*, a marker for resident T cells in the liver^31^, was enriched primarily in the C3 and C7 clusters. In HCC livers, the ChAT–GFP^+^ compartment contained a substantial number of C3 (*Cxcr6*^+^*Pdcd1*^+^) cells but was devoid of C7 (*Cxcr6*^+^*Pdcd1*^–^) cells (Fig. 2d). These C3 cells showed high expression of inhibitory immunoreceptors and exhaustion markers, such as *Pdcd1*, *Havcr2*, *Ptpn11*, *Lag3*, *Tox* and *Tigit*. These *Pdcd1*^+^ T cells also expressed *Cd247*, the gene encoding PD-L1, potentially providing an autologous ligand for PD-1 binding in addition to the PD-L1 expressed on APCs and HCC cells (Fig. 2f and Extended Data Fig. 2g). By contrast, C7 cells strongly expressed differentiation, cytotoxicity-related and other functional genes, such as *Il2ra*, *Il4*, *Il2*, *Fasl*, *Gzmb* and *Csf2*. These results suggest that *Chat* expression is associated with the appearance of dysfunctional *Pdcd1*^+^ T cells.

**Extended Data Fig. 3:**
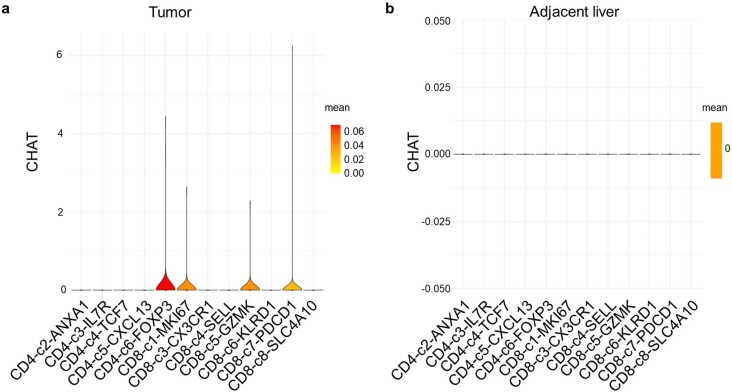
Identification of CHAT-expressing T cells in human HCC. **a,b** Violin plots showing the expression of *CHAT* in the indicated clusters of T cells that were isolated from tumor tissue (a) and adjacent liver tissue (b) of HCC patients. Data are from a published single-cell RNAseq dataset on immune cells of HCC patients (GSE140228). Related to Fig. 2. Source data

Examination of a published scRNA-seq dataset derived from immune cells isolated from individuals with HCC^32^ showed that ChAT-expressing T cells, including CD4^+^FOXP3^+^ T_reg_ cells, CD8^+^MKI67^+^ proliferating T cells, CD8^+^GZMK^+^ T cells and CD8^+^PDCD1^+^ T cells, were present in liver tumor tissues but not in adjacent normal liver tissues (Extended Data Fig. 3a,b). Thus, the existence and phenotypes of human ChAT-expressing T cells are consistent with our scRNA-seq data on T cells from mouse HCC, indicating that a similar induction program of ChAT-expressing T cells also occurs in human HCC.

### Cholinergic T_reg_ cells and PD-1^+^CD4^+^ T cells are induced in HCC

**Fig. 3:**
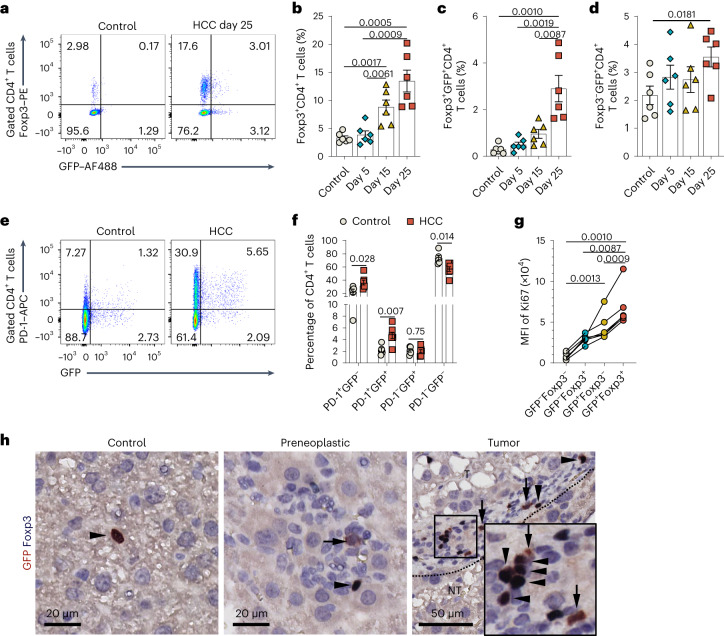
T_reg_ cells and PD-1^+^CD4^+^ T cells are overrepresented among HCC-induced ChAT-expressing T cells. **a**–**d**, Representative flow cytometric plots (**a**) and quantification (**b**–**d**) of the percentages of the indicated CD4^+^ T cell subsets expressing Foxp3 and/or GFP in livers that were isolated on the indicated days from control or HCC-bearing *Chat-GFP* reporter mice. In **b**–**d**, each dot represents an individual mouse (*n* = 6 per condition). Data are shown as mean ± s.e.m. *P* values were determined by unpaired, two-tailed *t*-test, and data are representative of three independent experiments. **e**,**f**, Representative flow cytometric plots (**e**) and quantification (**f**) of the percentages of the indicated CD4^+^ T cell subsets expressing PD-1 and/or GFP in livers from control (*n* = 5) or HCC-bearing (*n* = 7) *Chat-GFP* reporter mice. In **f**, each dot represents an individual mouse (*n* = 5 mice in control and *n* = 7 mice in HCC). Data are shown as mean ± s.e.m. *P* values were determined by unpaired, two-tailed *t*-test. Data represent the summary of three independent experiments. **g**, Quantitation of flow cytometric determination of Ki67 expression in ChAT–GFP^+^ and ChAT–GFP^–^ T_conv_ cells (Foxp3^–^) and T_reg_ cells (Foxp3^+^) in livers from HCC-bearing *Chat-GFP* mice. Each set of dots represents an individual mouse. *P* values were determined by paired two-tailed *t*-tests, and data are representative of three independent experiments. For **a**–**g**, the control group received transposase vector only. **h**, Representative histological sections of livers from the mice in **a**–**f** immunostained to detect Foxp3 and GFP in normal liver tissue (control; left), liver areas containing preneoplastic cells (middle) or HCC cells (right). Images are representative of three independent experiments. The inset box on the right shows a higher magnification view of the smaller boxed area. Arrowheads denote cells with dual Foxp3 and GFP immunoreactivity, and arrows denote cells with GFP immunoreactivity only; T, tumor tissue; NT, non-tumor tissue. Source data

To complement our scRNA-seq results, we profiled ChAT-expressing T cells in mouse HCC by flow cytometry. T_reg_ cell accumulation is an immune hallmark of liver cancer^33,34^. In our model, we observed a marked increase in Foxp3^+^ChAT–GFP^+^ T cells alongside expansion of T_reg_ cells during HCC development (Fig. 3a–c).

HCC livers also exhibited a significant increase in Foxp3^–^ChAT–GFP^+^CD4^+^ T cells (Fig. 3d), and PD-1^+^CD4^+^ T cells that were induced in HCC preferentially expressed ChAT–GFP (Fig. 3e,f). Furthermore, significantly higher Ki67 expression occurred on ChAT–GFP^+^Foxp3^+^ T_reg_ cells and Chat–GFP^+^Foxp3^–^ conventional T (T_conv_) cells (Fig. 3g), suggesting that proliferation underlies the induction of ChAT-expressing T cells in liver cancer.

Histologically, ChAT–GFP^+^Foxp3^+^ T_reg_ cells and ChAT–GFP^+^ T_conv_ cells accumulated in HCC tissue, in particular at the tumor border, and were also present in immune cell clusters associated with neoplastic hepatocytes (Fig. 3h).

### Tumor antigens drive cholinergic CD4^+^ T cell expansion

**Fig. 4:**
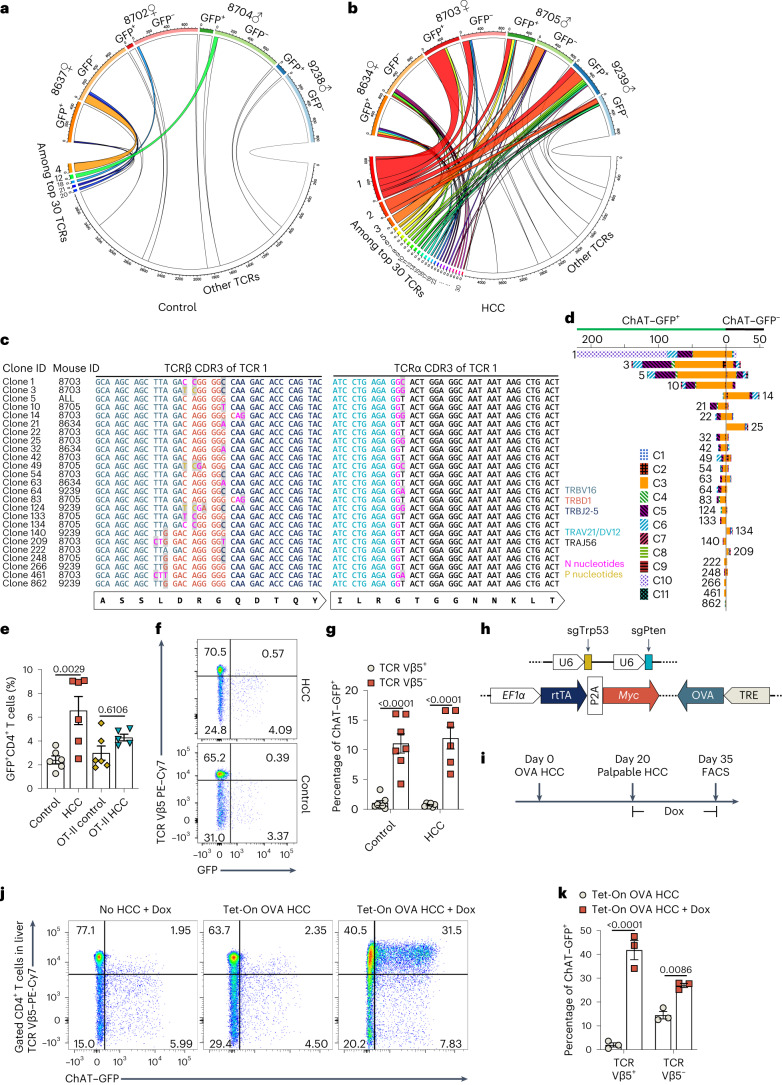
Tumor antigens drive clonal expansion of ChAT-expressing CD4^+^ T cells in HCC-bearing livers. **a**,**b**, Circos plots showing the distribution of TCR types among GFP^+^ and GFP^–^ T cells from control (**a**) and HCC-bearing (**b**) mice. T cells of the same TCR type share TCRα and TCRβ chains of the same amino acid sequences. The top 30 TCRs are numbered and highlighted with different colors. **c**, CDR3 sequences of clonotypes encoding TCR 1. *V*, *D* and *J* segments and N nucleotides and P nucleotides at V(D)J junctions are denoted with different colors. Nucleotides that are mismatched between clonotypes are shaded. **d**, Composition of ChAT–GFP^+^ and ChAT–GFP^–^ cells among T cells bearing the indicated TCR 1 clonotypes color-coded by cell clusters. Each bar represents an individual clonotype and is labeled with the clone ID as shown in **c**. Horizontal axis labels indicate cell numbers. **e**, Percentage of ChAT–GFP^+^ cells among CD4^+^ T cells from normal or HCC-bearing livers of *Chat-GFP* or *Chat-GFP*; OT-II mice (*n* = 6 in the control, HCC and OT-II control groups; *n* = 5 in the OT-II HCC group). Statistical significance was assessed by one-way analysis of variance (ANOVA) with Tukey’s multiple comparisons test, and data are representative of three independent experiments. **f**,**g**, Representative flow cytometry plots (**f**) and quantification (**g**) of percentages of ChAT–GFP^+^ cells among CD4^+^ T cells expressing TCR Vβ5^+^ (transgenic TCR) or TCR Vβ5^–^ (natural TCRs) in livers of *Chat-GFP*; OT-II mice (*n* = 7 in control; *n* = 6 in HCC). **h**, Plasmids for simultaneous CRISPR–Cas9-mediated deletion of *Trp53*/*Pten* plus overexpression of *Myc* and tetracycline-on (Tet-On) inducible OVA. **i**, Experimental protocol for inducing OVA expression. Dox was added to drinking water following palpable HCC onset; FACS, fluorescence-activated cell sorting. **j**,**k**, Representative flow cytometry plots (**j**) and quantification (**k**) of percentages of ChAT–GFP^+^ cells among CD4^+^ T cells expressing TCR Vβ5^+^ or TCR Vβ5^–^ in livers of mice that were left untreated or treated with Dox-containing drinking water following HCC onset. Data are the summary of two independent experiments (*n* = 3 mice per group). In **e**, **g** and **k**, each dot represents an individual mouse. Data are shown as mean ± s.e.m. In **g** and **k**, *P* values were determined by two-way ANOVA with Sidak’s multiple comparisons test. Source data

The proliferation characteristic of ChAT-expressing T cells in HCC livers led us to investigate the driving force behind their expansion. Single-cell TCR-seq revealed dominant TCR types across all animals (as defined by shared amino acid sequences for both the TCRα and TCRβ chains; Fig. 4a,b). Among the top 30 most prevalent TCR types, only 5 were present in control mice, while the remaining 25 were observed in their HCC-bearing littermates. Intriguingly, the prevalent TCRs in control mice preferentially belonged to ChAT–GFP^–^ T cells, while those in HCC-bearing mice were more commonly found on ChAT–GFP^+^ T cells (Fig. 4a,b). These results demonstrate a TCR-specific expansion of ChAT–GFP^+^ T cells in liver cancer.

The most dominant TCR type in HCC was denoted TCR 1, which was encoded by 25 clonotypes (as defined by shared mRNA sequences for both the TCRα and TCRβ chains). These clonotypes consisted of synonymous mRNA variants using the same combination of *V*, *D* and *J* genes. The variations arose from distinct junctions of the *V*, *D* and *J* segments occurring in different HCC-bearing mice, but the resulting amino acid sequences were identical due to codon redundancy (Fig. 4c). The majority of TCR 1 clonotypes were predominantly observed in ChAT–GFP^+^ cells (Fig. 4d). This TCR convergence across HCC-bearing mice suggests that TCRs specific for HCC antigens elicit the expansion of CD4^+^ T cells, particularly within the ChAT–GFP^+^CD4^+^ T cell compartment.

**Extended Data Fig. 4:**
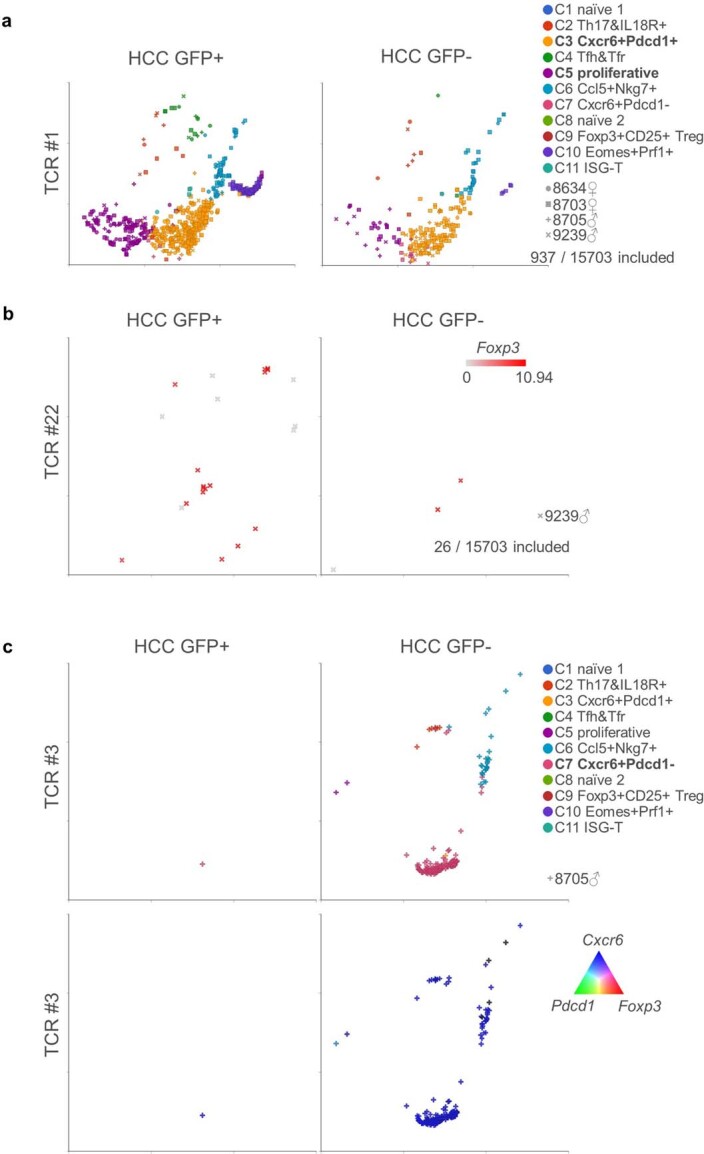
Expression of *Chat* by clonally expanded PD-1^+^ Tconvs and Foxp3^+^ Tregs in HCC. **a** UMAP representation of Chat-GFP^+^ and Chat-GFP^-^ CD4^+^ T cells bearing TCR #1 induced in HCC, color-coded by cell clusters and shape-coded by mouse ID. **b** UMAP representation of Chat-GFP^+^ and Chat-GFP^-^ CD4^+^ T cells bearing TCR #22 induced in HCC, color-coded by expression level of *Foxp3* and shape-coded by mouse ID. **c** UMAP representation of Chat-GFP^+^ and Chat-GFP^-^ CD4^+^ T cells bearing TCR #3 induced in HCC, color-coded by cell clusters (upper) and expression levels of *Cxcr6*, *Pdcd1*, and *Foxp3* (lower), and shape-coded by mouse ID. Related to Fig. 4.

We next characterized the cells bearing particular TCR types. T cells carrying TCR 1 were mainly from the C3 cluster that harbored ChAT-expressing *Pdcd1*^+^ T_conv_ cells (Fig. 4d and Extended Data Fig. 4a). T cells carrying TCR 22 were chiefly ChAT-expressing *Foxp3*^+^ T_reg_ cells (Extended Data Fig. 4b). These results reveal an accumulation of ChAT-expressing, clonally expanded PD-1^+^ T_conv_ cells and Foxp3^+^ T_reg_ cells in HCC. Pertinently, clonal expansion of T_reg_ cells has also been revealed by TCR usage analysis at the single-cell level in human HCC^34^. Notably, T cells bearing TCR 3 were exclusively ChAT–GFP^–^ cells in HCC (Fig. 4b) and belonged primarily to cluster C7 (*Cxcr6*^+^*Pdcd1*^–^; Extended Data Fig. 4c). Among the 25 clonotypes of TCR 1, clones 14 and 25 were predominantly C3 cells but ChAT–GFP^–^, although they carried the same TCR and shared a similar composition of cell clusters with many other ChAT–GFP^+^ clonotypes of TCR 1 (Fig. 4c,d). We postulate that this clonal divergence in ChAT expression likely reflects the complexity of the tumor microenvironment.

To determine the role of tumor antigens in the induction of ChAT-expressing T cells, we crossed *Chat-GFP* mice with chicken ovalbumin (OVA)-specific TCR transgenic OT-II mice. First, we subjected *Chat-GFP*; OT-II mice to standard HCC induction, which does not involve OVA expression. We found that ChAT–GFP^+^ T cells did not become significantly elevated, and most were CD4^+^ T cells expressing a natural repertoire of TCRs that were Vβ5^–^ (Fig. 4e–g).

Next, we used OVA to mimic a tumor antigen and devised a vector allowing the inducible expression of cytosolic OVA with constitutive expression of *Myc*. We introduced this vector alongside the CRISPR *Trp53*/*Pten* deletion vector into *Chat-GFP*; OT-II mice (Fig. 4h). When HCC tumors were palpable, doxycycline (Dox) was administered to these mice through their drinking water to induce OVA expression (Fig. 4i). When we compared ChAT expression in Dox-treated and untreated mice, we found that about 40% of OVA-specific (TCR Vβ5^+^) CD4^+^ T cells expressed ChAT after OVA induction in HCC (Fig. 4j,k). In untreated mice, only about 2% of Vβ5^+^CD4^+^ T cells expressed ChAT, comparable to the percentage in *Chat-GFP*; OT-II mice bearing OVA^–^ HCC (Fig. 4f,g,j,k). Interestingly, we also observed an elevated percentage of ChAT–GFP^+^ cells among CD4^+^ T cells carrying natural TCRs (TCR Vβ5^–^) (Fig. 4j,k), suggesting that ChAT expression in T cells can also be induced through ‘antigen spreading’^35^.

**Extended Data Fig. 5:**
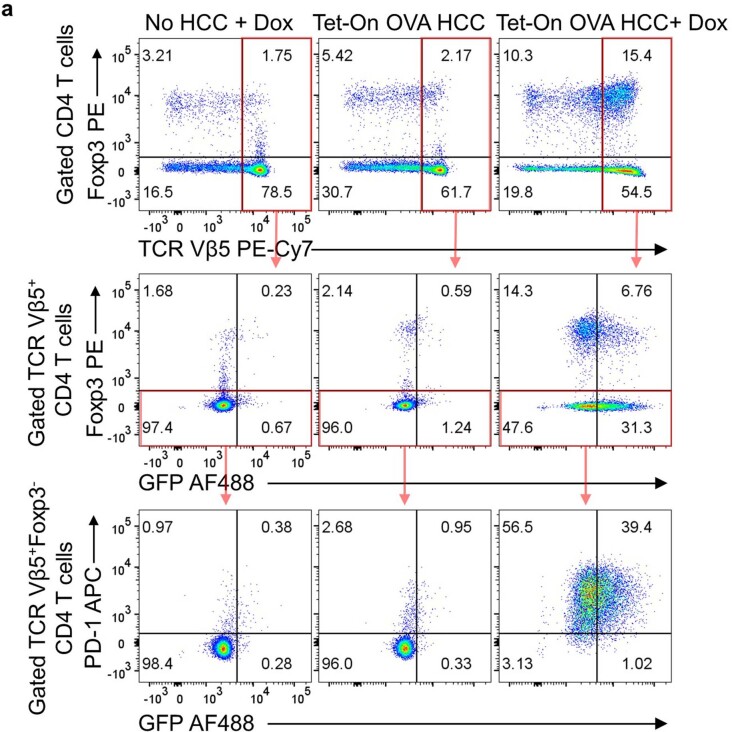
Induction of OVA specific Chat-GFP^+^ Tregs and PD-1^+^ Tconvs in HCC with inducible OVA expression. **a** Representative flow cytometry plots showing the expression of Foxp3 and PD-1 in OVA-specific Chat-GFP^+^CD4^+^ T cells in livers of mice that were left untreated or treated with Dox-containing drinking water to induce OVA expression following HCC onset, representing two independent experiments. Related to Fig. 4.

We further characterized the OVA-specific ChAT^+^ T cells in our HCC model with inducible OVA expression and found that Dox-mediated activation of OVA expression markedly induced T_reg_ cells, especially among OVA-specific T cells (Vβ5^+^; Extended Data Fig. 5a). There was also an induction of ChAT–GFP^+^ T cells among both Foxp3^+^ T_reg_ cells and Foxp3^–^ T_conv_ cells harboring OVA-specific TCRs (Extended Data Fig. 5a). PD-1 was highly expressed by these OVA-specific T_conv_ cells, and ~40% of these cells were ChAT–GFP^+^ (Extended Data Fig. 5a). Taken together, these data confirm that tumor antigens can induce ChAT-expressing T_reg_ cells and PD-1^+^ T_conv_ cells and demonstrate that the expansion of ChAT-expressing T cells in liver cancer depends on TCR activation by tumor antigens.

### Ablation of *Chat* in T cells dampens HCC immunosurveillance

**Fig. 5:**
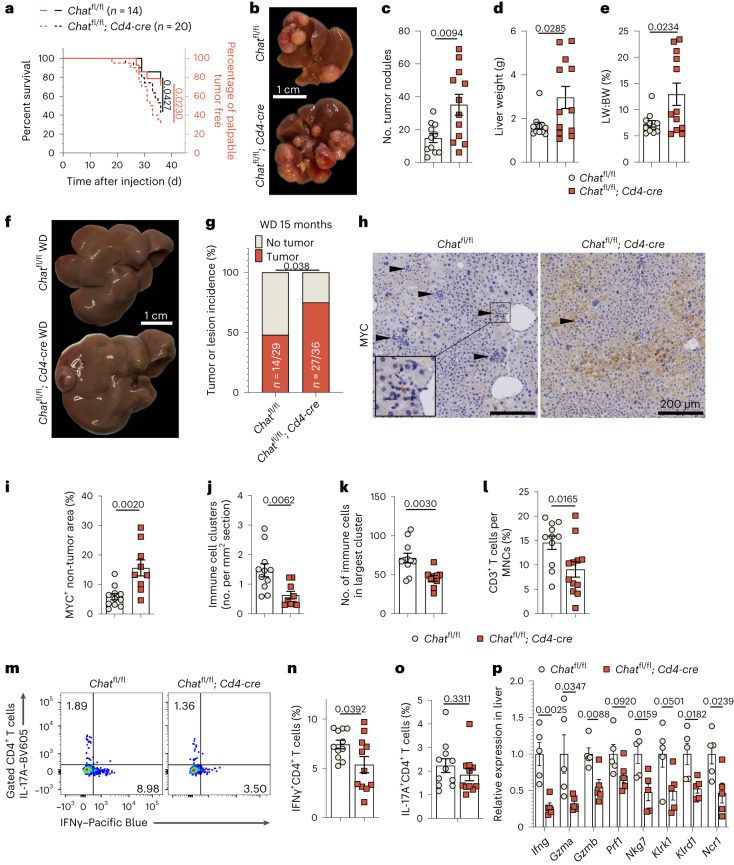
Ablation of *Chat* in T cells inhibits the immunosurveillance of liver cancer in mice. **a**, Curves showing latency to palpable HCC development (left, black lines) and survival to humane endpoint (right, red lines) of *Chat*^fl/fl^ (*n* = 14) and *Chat*^fl/fl^; *Cd4-cre* (*n* = 20) mice. *P* values were determined by log-rank (Mantel–Cox) test, and data are representative of two independent experiments. **b**–**e**, Representative images (**b**), numbers of tumor nodules (**c**), liver weights (**d**) and ratios of liver weight (LW) to body weight (BW; **e**) of mice on day 35 of HCC induction. For **c**–**e**, *n* = 11 mice in the *Chat*^fl/fl^ group and *n* = 12 mice in *Chat*^fl/fl^; *Cd4-cre* group; data are representative of five independent experiments. **f**,**g**, Representative images (**f**) and quantification (**g**) of tumor incidence in mice fed for 15 months on a Western diet (WD). *P* values were determined by Fisher’s exact test. **h**,**i**, Representative histological sections (**h**) and quantification (**i**) of immunostaining to detect MYC^+^ preneoplastic cells in non-tumor areas of liver sections from the mice in **b**–**e**. In **i**, *n* = 11 *Chat*^fl/fl^ mice and *n* = 9 *Chat*^fl/fl^; *Cd4-cre* mice. The inset box in **h** shows a higher-magnification view of the smaller boxed area. Arrowheads indicate immune cell clusters. **j**,**k**, Quantification of the number (**j**) and size (**k**) of immune cell clusters in hematoxylin and eosin (H&E)-stained sections of livers from the mice in **b**–**e** (*n* = 11 *Chat*^fl/fl^ mice and *n* = 9 *Chat*^fl/fl^; *Cd4-cre* mice). **l**, Percentage of CD3^+^ T cells among MNCs isolated from HCC-bearing livers as assessed by flow cytometry (*n* = 11 *Chat*^fl/fl^ mice and *n* = 12 *Chat*^fl/fl^; *Cd4-cre* mice); data are representative of three independent experiments. **m**–**o**, Representative flow cytometry plots (**m**) and quantification of IFNγ^+^CD4^+^ (**n**) and IL-17A^+^CD4^+^ T cells (**o**) in HCC-bearing livers (*n* = 11 mice per group); data are representative of two independent experiments. **p**, Quantitative PCR (qPCR) determination of mRNA levels (relative to *Actb*) of the indicated cytotoxicity genes and NK cell marker genes in HCC-bearing livers (*n* = 5 mice per group); data are representative of two independent experiments. For **c**–**e**, **i**–**l** and **n**–**p**, each dot represents an individual mouse. Data are shown as mean ± s.e.m., and *P* values were determined by unpaired, two-tailed *t*-tests. Source data

To determine the role of ChAT-expressing T cells in the onset of liver cancer, we deleted *Chat* specifically in T cells by crossing mice carrying the conditional *Chat*^fl^ allele with mice expressing the *Cd4-cre* transgene, thereby obtaining *Chat*^fl/fl^; *Cd4-cre* progeny. When we subjected these animals (and *Chat*^fl/fl^ controls) to HCC induction, we found that *Chat*^fl/fl^; *Cd4-cre* mice developed liver cancer much faster than their *Chat*^fl/fl^ littermates (Fig. 5a). The numbers of tumor nodules and liver weights were also significantly increased in *Chat*^fl/fl^; *Cd4-cre* mice (Fig. 5b–e).

To further substantiate the role of ChAT-expressing T cells in liver tumorigenesis, we used an alternative disease model in which *Chat*^fl/fl^; *Cd4-cre* and *Chat*^fl/fl^ mice were fed long term on a Western diet (high fat, high cholesterol and high sugar) to induce non-alcoholic steatohepatitis (NASH). NASH sets the stage for liver cirrhosis, which eventually progresses to spontaneous HCC^8,36^. After 15 months on the Western diet, we found that the incidence of NASH-derived HCC was significantly higher in mice bearing T cells lacking *Chat* (Fig. 5f,g). These consistent results from two models of HCC development establish that a deficiency of *Chat* in T cells renders mice susceptible to liver tumorigenesis.

**Extended Data Fig. 6:**
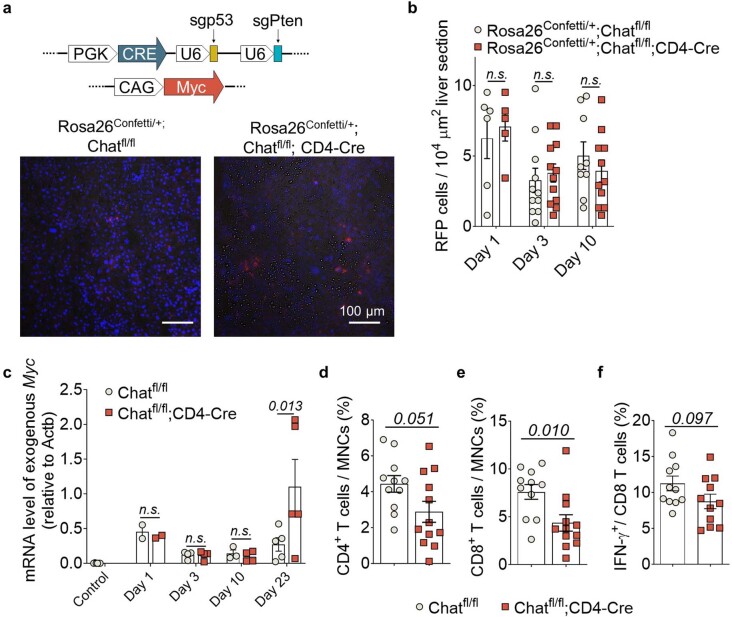
Analysis of the efficiency of vector delivery and T cell activities in HCC. **a,b** Representative fluorescence microscopy images (a), and quantification of the proportion of hepatocytes positive for the *Rosa26*^*Confetti*^ RFP marker (b), in liver sections from *Rosa26*^*Confetti*/+^;*Chat*^*fl/fl*^ and *Rosa26*^*Confetti*/+^*;Chat*^*fl/fl*^*;CD4-Cre* mice on the indicated days following plasmid injection. In (b), each dot represents a microscopy image (n=6, 11, and 9 acquired on days 1, 3, and 10 from *Rosa26*^*Confetti*/+^;*Chat*^*fl/fl*^ mice; n=5, 12, and 11 acquired on days 1, 3, and 10 from *Rosa26*^*Confetti*/+^*;Chat*^*fl/fl*^*;CD4-Cre* mice. Images were collected from 2-4 mice/group). Data are the mean ± SEM. P values were determined by two-way ANOVA with Tukey’s multiple comparisons test. **c** qPCR determination of transposon-derived *Myc* mRNA expression (relative to *Actb*) in livers of *Chat*^*fl/fl*^ and *CD4-Cre;Chat*^*fl/fl*^ mice at the indicated time points following HCC induction. The forward primer used was within the *Myc* ORF, with the reverse primer in the transposon vector. Each dot represents an individual mouse (n=6 *Chat*^*fl/fl*^ mice in Control group, and n=2, 4, 3 and 5 *Chat*^*fl/fl*^ mice on days 1, 3, 10, and 23; n=2, 4, 4, and 5 *Chat*^*fl/fl*^*;CD4-Cre* mice on days 1, 3, 10 and 23). Data are the mean ± SEM. P values were determined by two-way ANOVA with Sidak’s multiple comparisons test. **d,e** Percentages of CD4^+^ T cells (d) and CD8^+^ T cells (e) among mononuclear cells (MNCs) isolated from HCC-bearing livers of *Chat*^*fl/fl*^ and *CD4-Cre;Chat*^*fl/fl*^ mice as assessed by flow cytometry, representative of three independent experiments. Each dot represents an individual mouse (n=11 mice in *Chat*^*fl/fl*^ and n=12 mice in *Chat*^*fl/fl*^*;CD4-Cre*). Data are the mean ± SEM. P values were determined by unpaired, two-tailed t-test. **f** Percentage of IFN-γ^+^ CD8^+^ T cells in HCC-bearing livers of *Chat*^*fl/fl*^ and *Chat*^*fl/fl*^*;CD4-Cre* mice as analyzed by flow cytometry. Each dot represents an individual mouse. Each dot represents an individual mouse (n=11 mice in each group). Data are the mean ± SEM. P values were determined by unpaired, two-tailed t-test. Related to Fig. 5. Source data

Before overt tumor nodules appeared in our HCC model, preneoplastic cells could be identified by their high MYC expression (Fig. 5h), reflecting successful transfection and oncogene expression. The proportion of preneoplastic hepatocytes showing high MYC expression was significantly elevated in livers of *Chat*^fl/fl^; *Cd4-cre* mice, and this increase was not due to a difference in vector delivery (Fig. 5h,i and Extended Data Fig. 6a–c). Immune cells are critical for clearing preneoplastic cells from the liver^5^. In *Chat*^fl/fl^ mice, we observed immune cell clusters around MYC-expressing preneoplastic cells (Fig. 5h). These clusters were reduced in frequency and size in *Chat*^fl/fl^; *Cd4-cre* mice (Fig. 5h,j,k), suggesting a defect in immunosurveillance of preneoplastic cells. Accordingly, T cell infiltration into HCC-bearing livers was significantly decreased in the absence of *Chat* (Fig. 5l and Extended Data Fig. 6d,e).

IFNγ, a hallmark of the type 1 helper T cell immune response, has a pivotal function in antitumor immunity^37^. We found that IFNγ production by HCC-associated T cells was decreased in *Chat*^fl/fl^; *Cd4-cre* mice (Fig. 5m–p and Extended Data Fig. 6f). In addition to adaptive immune responses, innate cytotoxic NK cells play a crucial role in antitumor immune response in HCC^38,39^. We observed that mRNA levels of genes encoding IFNγ, granzymes and perforin, which are cytotoxic effectors shared by cytotoxic T cells and NK cells, were significantly decreased in *Chat*^fl/fl^; *Cd4-cre* mice (Fig. 5p, left). These deficits correlated with reduced expression of NK cell marker genes (Fig. 5p, right). Therefore, both adaptive and innate antitumor immune responses are hampered by the ablation of *Chat* in T cells.

### T_reg_ cells blunt HCC immunosurveillance in *Chat*^fl/fl^; *Cd4-cre* mice

**Extended Data Fig. 7:**
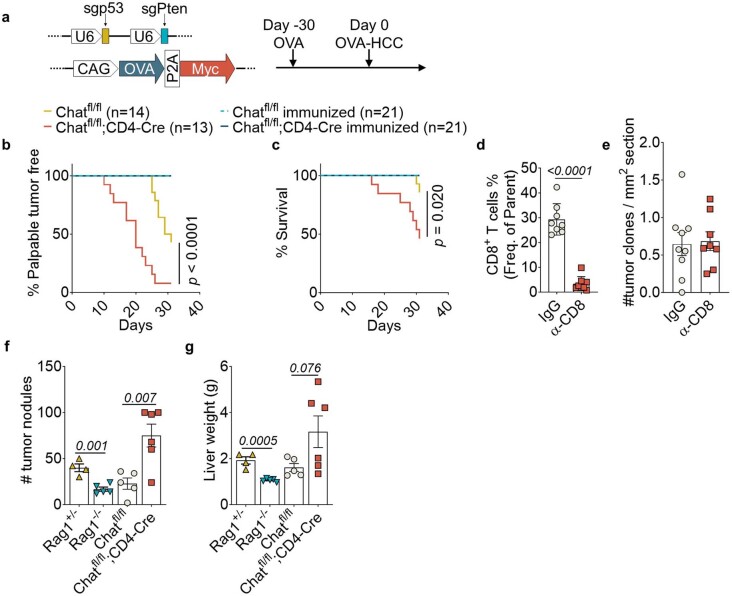
Delineating the role of the adaptive immune response in HCC development. **a** Schematic diagrams of (left) the plasmids used to induce simultaneous CRISPR/Cas9-mediated deletion of *Trp53* and *Pten* plus overexpression of *Myc* and chicken ovalbumin (OVA), and (right) the experimental protocol for OVA immunization and OVA-HCC induction in mice. See main text for details. **b,c** Curves showing the latency to palpable HCC development (b), and survival to humane endpoint (c), of *Chat*^*fl/fl*^ and *Chat*^*fl/fl*^*;CD4-Cre* mice that were left unimmunized, or immunized as depicted in panel (a), and subjected to OVA-HCC induction (n=14, 13, 21, and 21 mice in the *Chat*^*fl/fl*^, *Chat*^*fl/fl*^*;CD4-Cre*, *Chat*^*fl/fl*^ immunized and *Chat*^*fl/fl*^*;CD4-Cre* immunized groups, respectively). P values were determined by log-rank test. **d,e** Percentage of CD8^+^ T cells among MNCs (d), and number of tumor nodules observed in histological sections (e), in HCC-bearing livers of mice treated with anti-CD8 antibodies (α-CD8) or IgG control. Each dot represents an individual mouse (n=8 mice per group). Data are the mean ± SEM. In (d), P value was determined by unpaired, two-tailed t-test. **f,g** Number of HCC tumor nodules (f), and weights (g) of livers from mice of the indicated genotypes on day 23 of HCC induction. Each dot represents an individual mouse (n=4, 5, 5, and 6 mice in the *Rag1*^*+/-*^, *Rag1*^*-/-*^, *Chat*^*fl/fl*^ and *Chat*^*fl/fl*^*;CD4-Cre* groups, respectively). Data are the mean ± SEM. P values were determined by paired, two-tailed t-test. Related to Fig. 6. Source data

To delve more deeply into the mechanism of antitumor immunity mediated by ChAT-expressing T cells, we devised a vector mediating the expression of OVA alongside MYC. We introduced this vector plus our *Trp53*/*Pten* deletion vector into OVA-immunized and non-immunized mice (Extended Data Fig. 7a). In non-immunized mice, tumor progression to endpoint was significantly accelerated by *Chat* ablation in T cells (Extended Data Fig. 7b,c), consistent with our previous observations (Fig. 5a). However, OVA-immunized *Chat*^fl/fl^ and *Chat*^fl/fl^; *Cd4-cre* mice were equally protected against HCC development (Extended Data Fig. 7b,c). These results showed that the antitumor immune response elicited by a potently immunogenic tumor antigen was not compromised in the absence of ChAT in T cells. In addition, depletion of cytotoxic T lymphocytes (CTLs) by treatment with anti-CD8 had little effect on liver tumor burden (Extended Data Fig. 7d,e). Thus, CTLs do not play a non-redundant role in the immunosurveillance of liver cancer in this setting.

To determine if loss of adaptive immune cells in general would compromise antitumor immunity in our model, we compared the onset of HCC in lymphocyte-deficient *Rag1*^–/–^ mice to that in *Chat*^fl/fl^; *Cd4-cre* mice. In contrast to the significantly increased tumor burden in *Chat*^fl/fl^; *Cd4-cre* mice, HCC progression was not exacerbated in *Rag1*^–/–^ mice but instead was alleviated (Extended Data Fig. 7f,g). We reasoned that this unexpected observation could be attributed to the absence of T_reg_ cells and dysfunctional effector T cells in *Rag1*^–/–^ mice. This loss of control by adaptive immune cells (especially T_reg_ cells) causes *Rag1*^–/–^ mice to exhibit an excessive innate immune response by NK cells^40^. The fact that HCC progression was aggravated in NSG mice (which lack both adaptive immune cells and NK cells; Fig. 1h) bolsters our contention that the antitumor activity of NK cells in our model is curbed by adaptive immune cells, particularly T_reg_ cells.

**Fig. 6:**
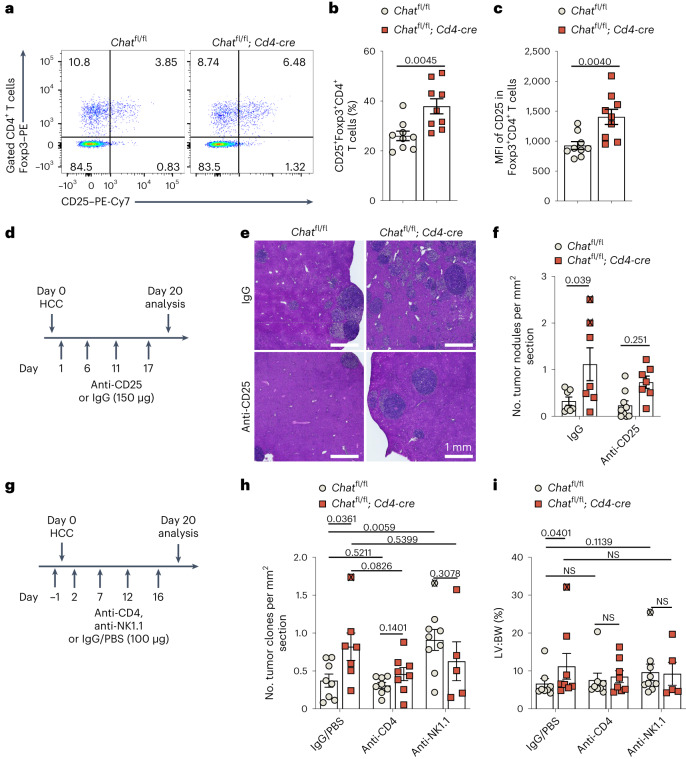
T cell-specific loss of *Chat* causes alterations to T_reg_ cells that are linked to compromised antitumor immunity. **a**–**c**, Representative flow cytometry plots (**a**), quantification of the percentages of Foxp3^+^CD4^+^ T cells expressing CD25 (**b**) and the CD25 mean fluorescent intensity (MFI; **c**) in HCC-bearing livers from *Chat*^fl/fl^ and *Chat*^fl/fl^; *Cd4-cre* mice on day 35 of HCC induction (*n* = 9 mice per group). *P* values were determined by unpaired, two-tailed *t*-test; data are representative of two independent experiments. **d**, Experimental protocol used to deplete CD25-expressing T_reg_ cells during HCC induction. **e**,**f**, Representative low-magnification histological images of H&E-stained sections (**e**) and quantification of numbers of tumor nodules per mm^2^ in these sections (**f**) of livers from *Chat*^fl/fl^ and *Chat*^fl/fl^; *Cd4-cre* mice treated as in **d**. In **f**, *n* = 7 mice in the *Chat*^fl/fl^ + IgG, *Chat*^fl/fl^ + anti-CD25 and *Chat*^fl/fl^; *Cd4-cre* + anti-CD25 groups, and *n* = 9 mice in the *Chat*^fl/fl^ + anti-CD25 group. *P* values were determined by two-way ANOVA with Tukey’s multiple comparisons tests. The ‘X’ symbols indicate animals that reached the humane endpoint before day 20. **g**, Experimental protocol used to deplete CD4^+^ T cells and NK cells during HCC induction. Each mouse received 100 μg of depleting antibody per injection. Mice in the control group received either 100 μg of rat IgG or PBS. **h**,**i**, Quantification of numbers of tumor nodules per mm^2^ in H&E-stained sections (**h**) and ratios of liver weight to body weight (**i**) of *Chat*^fl/fl^ and *Chat*^fl/fl^; *Cd4-cre* mice treated as in **g**. The ‘X’ symbols indicate animals that reached the humane endpoint before day 20. In **h**, *n* = 8 mice in the *Chat*^fl/fl^ + IgG/PBS and anti-CD4 groups; *n* = 7 mice in the *Chat*^fl/fl^; *Cd4-cre* + IgG/PBS group; *n* = 9 mice in the *Chat*^fl/fl^ + anti-NK1.1 group; and *n* = 5 mice in the *Chat*^fl/fl^; *Cd4-cre* + anti-NK1.1 group. In **i**, *n* = 8 mice in the IgG/PBS and anti-CD4 groups; *n* = 9 mice in the *Chat*^fl/fl^ + anti-NK1.1 group; and *n* = 5 mice in the *Chat*^fl/fl^; *Cd4-cre* + anti-NK1.1 group. In **b**, **c**, **f**, **h** and **i**, each dot represents an individual mouse. Data are shown as means ± s.e.m. *P* values in **h** were determined by paired, two-tailed *t*-tests. *P* values in **i** were determined by two-tailed Mann–Whitney test because the *Chat*^fl/fl^ group did not pass the normality test; NS, not significant. Source data

**Extended Data Fig. 8:**
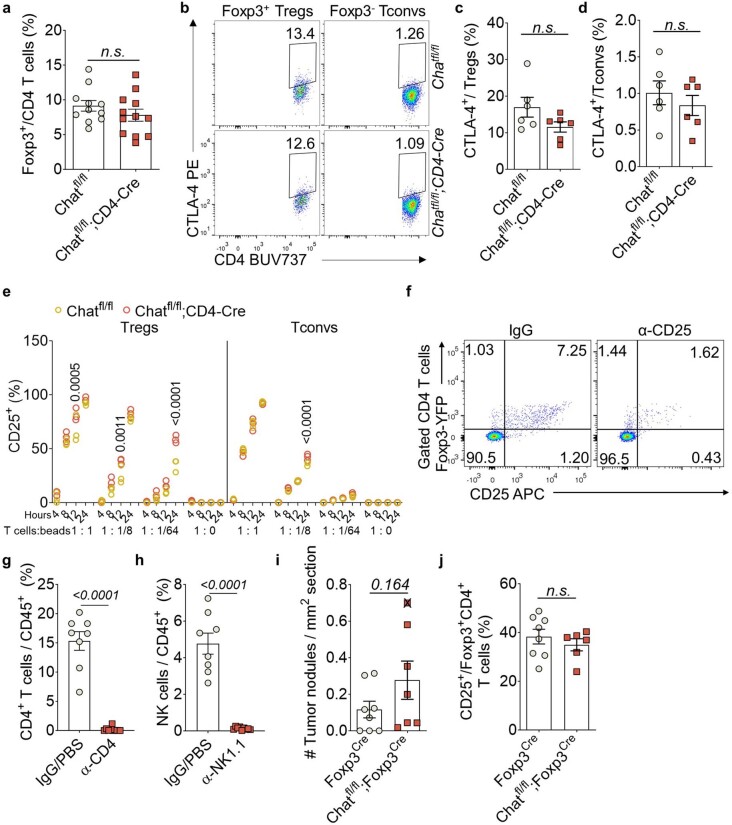
Delineating the effects of *Chat* on Tregs in HCC. **a** Percentage of Foxp3^+^ Tregs among CD4^+^ T cells in HCC-bearing livers of *Chat*^*fl/fl*^ and *CD4-Cre;Chat*^*fl/fl*^ mice, representative of three independent experiments. Each dot represents an individual animal (n=11 mice in *Chat*^*fl/fl*^ and n=12 mice in *Chat*^*fl/fl*^*;CD4-Cre*). **b-d** Representative flow cytometry plots (b), and quantification of the percentages of CTLA-4^+^ cells among Tregs (c) and Tconvs (d), in HCC-bearing livers from *Chat*^*fl/fl*^ and *Chat*^*fl/fl*^*;CD4-Cre* mice on day 16 of HCC induction. In (c) and (d), each dot represents an individual mouse (n=6 mice per group). **e** Quantification of the percentages of induced CD25^+^ cells among Foxp3^+^CD4^+^ Tregs and Foxp3^-^CD4^+^ Tconvs isolated from purified splenic CD4^+^CD25^-^ T cells of *Chat*^*fl/fl*^ and *Chat*^*fl/fl*^*;CD4-Cre* mice. CD4^+^CD25^-^ T cells were enriched with negative selection microbeads and stimulated with the indicated ratios of anti-CD3/28 microbeads. CD25 expression was analyzed by flow cytometry at the indicated time points. P values were determined by unpaired, two-tailed t-test, three replicates for each condition, representative of two independent experiments. **f** Representative flow cytometric plots of CD25^+^ Foxp3^+^ CD4^+^ Tregs in blood of *Foxp3-YFP* mice that were treated with either anti-CD25 antibody (α-CD25) or control IgG. Data are representative of 2 mice/group analyzed on day 18 of antibody treatment. **g,h** Percentage of CD4^+^ T cells (g) and NK cells (h) among MNCs from HCC-bearing livers of mice treated with anti-CD4 antibodies (α-CD4), anti-NK1.1 antibodies (α-NK1.1) or IgG/PBS control. Each dot represents an individual mouse (n=8 mice in the IgG/PBS group; n=9 mice in α-CD4 and α-NK1.1 groups). **i,j** Quantification of numbers of tumor nodules/mm^2^ in H&E-stained sections (i), and percentages of CD25^+^ Tregs in HCC-bearing livers (j), of *Foxp3*^Cre^ and *Chat*^*fl/fl*^;*Foxp3*^*Cre*^ mice on day 22 of HCC induction. Each dot represents an individual mouse. In (i), n=8 mice in the *Foxp3*^Cre^ group and n=7 mice in the *Chat*^*fl/fl*^;*Foxp3*^*Cre*^ group. In (j), n=8 mice in *Foxp3*^Cre^ and n=6 mice in *Chat*^*fl/fl*^;*Foxp3*^*Cre*^. In (a), (c), (d), and (g-j), data are the mean ± SEM. P values were determined by unpaired, two-tailed t-test. Related to Figs. 6 and 7. Source data

To investigate our hypothesis that the accelerated tumor onset linked to T cell-specific *Chat* ablation could be due to suppression of antitumor responses by elevated T_reg_ cell activity and/or T_conv_ cell dysfunction, we examined how T_reg_ cells modulate the antitumor activity of ChAT-expressing T cells. We observed no difference in the abundance of Foxp3^+^ T_reg_ cells in liver tumors of *Chat*^fl/fl^ and *Chat*^fl/fl^; *Cd4-cre* mice (Extended Data Fig. 8a). However, CD25 expression by Foxp3^+^ T_reg_ cells in *Chat*^fl/fl^; *Cd4-cre* mice was substantially increased compared to in control mice (Fig. 6a–c), whereas CTLA-4 expression levels were similar (Extended Data Fig. 8b–d). We then purified CD25^–^CD4^+^ T cells from spleens of *Chat*^fl/fl^ and *Chat*^fl/fl^; *Cd4-cre* mice and analyzed CD25 induction following TCR stimulation by anti-CD3/CD28 beads. CD25 expression in Foxp3^+^ T_reg_ cells from *Chat*^fl/fl^; *Cd4-cre* mice was significantly higher than in T_reg_ cells from *Chat*^fl/fl^ mice (Extended Data Fig. 8e). This induction of CD25 by TCR activation was much stronger on T_reg_ cells than on T_conv_ cells (Extended Data Fig. 8e). Therefore, TCR-induced expression of CD25 in T_reg_ cells is modulated by ChAT in T cells. When we used anti-CD25 to deplete CD25-expressing T_reg_ cells (Fig. 6d and Extended Data Fig. 8f), we observed that the enhanced tumor burden in *Chat*^fl/fl^; *Cd4-cre* mice was partially decreased (Fig. 6e,f). Together, these results point toward the involvement of T_reg_ cells in the suppression of antitumor immune responses in *Chat*^fl/fl^; *Cd4-cre* mice.

CD25-expressing T_reg_ cells inhibit the antitumor activities of cytotoxic T cells and NK cells^41^. *Chat*^fl/fl^; *Cd4-cre* mice showed significantly increased expression of CD25 on T_reg_ cells (Fig. 6a–c) and reduced numbers of IFNγ^+^CD4^+^ T cells and NK cells (Fig. 5m–p). When we removed CD4^+^ T cells during HCC induction using anti-CD4 (Fig. 6g and Extended Data Fig. 8g), we observed that CD4^+^ T cell depletion did not affect HCC progression in *Chat*^fl/fl^ mice but tended to reduce it in *Chat*^fl/fl^; *Cd4-cre* mice (Fig. 6h,i). This result suggested that the collective functions of T_reg_ cells and T_conv_ cells in HCC are neutral in *Chat*^fl/fl^ mice, whereas *Chat* deletion in T cells tilts the balance toward HCC promotion. Because CD4^+^ T cell depletion abolished the difference in HCC development between *Chat*^fl/fl^ mice and *Chat*^fl/fl^; *Cd4-cre* mice, the independent contributions to HCC of other ChAT-expressing T lineage cells, including CD8^+^ T cells and NKT cells, appear to be minor. When we used anti-NK1.1 to deplete NK cells from our model (Fig. 6g and Extended Data Fig. 8h), HCC development was promoted in *Chat*^fl/fl^ mice but not in *Chat*^fl/fl^; *Cd4-cre* mice, essentially eliminating the differences in tumor progression (Fig. 6h,i). Thus, NK cells are indispensable for HCC immunosurveillance in our model, and the antitumor functions of NK cells are impeded in the absence of cholinergic T cells.

### ChAT loss in T cells exacerbates T cell dysfunction in HCC

**Fig. 7:**
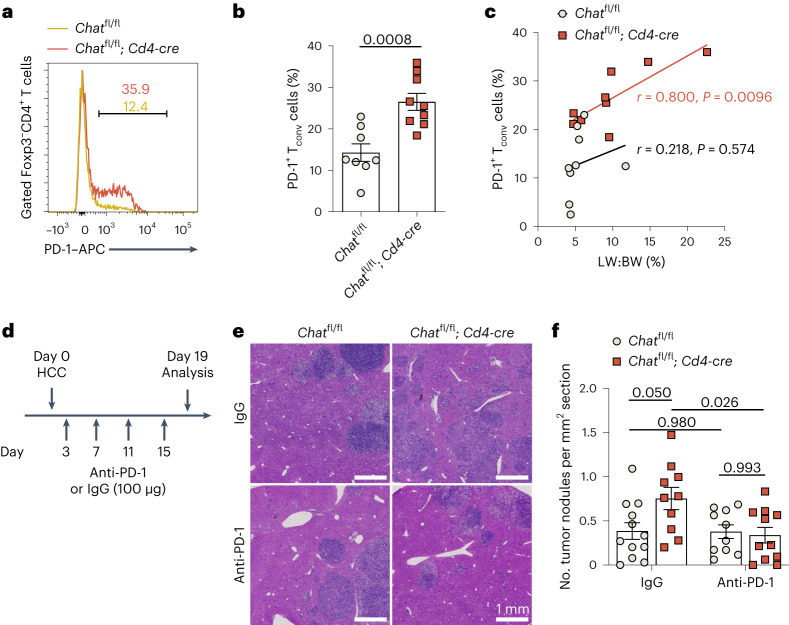
PD-1 inhibitory activity is unleashed in the absence of *Chat* in T cells in HCC. **a**,**b**, Representative flow cytometry histogram overlay plot (**a**) and quantification of the percentages of Foxp3^–^CD4^+^ T_conv_ cells expressing PD-1 (**b**) in HCC-bearing livers from *Chat*^fl/fl^ and *Chat*^fl/fl^; *Cd4-cre* mice. Each dot represents an individual mouse (*n* = 8 *Chat*^fl/fl^ mice and *n* = 9 *Chat*^fl/fl^; *Cd4-cre* mice). Data are shown as mean ± s.e.m. The *P* value was determined by unpaired, two-tailed *t*-test. **c**, Correlation of PD-1 expression in T_conv_ cells with HCC grade in *Chat*^fl/fl^ and *Chat*^fl/fl^; *Cd4-cre* mice. HCC grade is represented by the ratio of liver weight to body weight. *P* values were determined by two-tailed Pearson correlation. **d**, Experimental protocol used for PD-1 blockade in vivo in *Chat*^fl/fl^ and *Chat*^fl/fl^; *Cd4-cre* mice subjected to standard HCC induction. **e**,**f**, Representative low-magnification histological images of H&E-stained sections (**e**) and quantification of numbers of tumor nodules per mm^2^ in these sections (**f**) of livers from *Chat*^fl/fl^ and *Chat*^fl/fl^; *Cd4-cre* mice treated as in **d**. In **f**, each dot represents an individual mouse (*n* = 12 mice in the *Chat*^fl/fl^ + IgG group, *n* = 10 mice in the *Chat*^fl/fl^ + anti-PD-1 and *Chat*^fl/fl^; *Cd4-cre* + IgG groups, and *n* = 11 mice in the *Chat*^fl/fl^ + anit-PD-1 group). Data are shown as mean ± s.e.m. *P* values were determined by two-way ANOVA with Tukey’s multiple comparisons test. Source data

**Extended Data Fig. 9:**
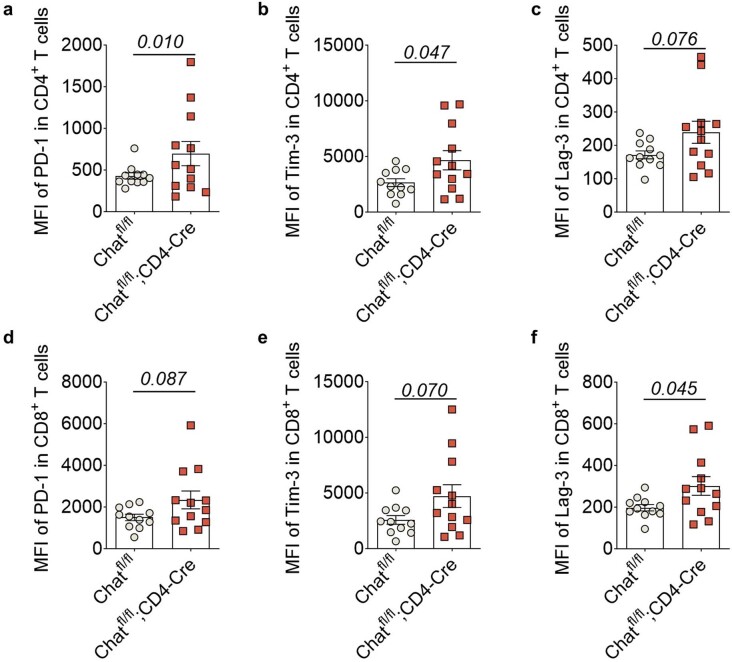
Elucidating the effects of *Chat* on inhibitory immunoreceptor expression by T cells in HCC. **a-f** Expression levels of the immune inhibitory receptors PD-1, Tim-3 and Lag-3 in CD4^+^ T cells (a-c), and in CD8^+^ T cells (d-f), from HCC-bearing livers of *Chat*^*fl/fl*^ and *Chat*^*fl/fl*^;*CD4-Cre* mice on day 35 of HCC induction. Each dot represents an individual mouse (n=11 mice in *Chat*^*fl/fl*^ and n=12 mice in *Chat*^*fl/fl*^*;CD4-Cre*). Data are the mean ± SEM. P values were determined by unpaired, two-tailed t-test. Related to Fig. 7. Source data

To investigate the role of ChAT specifically in T_reg_ cells, we crossed *Chat*^fl/fl^ mice with *Foxp3*^Cre^ mice and induced HCC in *Foxp3*^Cre^ and *Foxp3*^Cre^; *Chat*^fl/fl^ littermates. We found that deleting *Chat* only in T_reg_ cells had a milder effect on HCC progression than did deleting *Chat* in all T cells (Extended Data Fig. 8i,j), suggesting that ChAT-expressing T_conv_ cells are indispensable for a full-fledged anti-HCC immune response. ChAT-expressing T_conv_ cells induced in HCC are primarily PD-1^+^ T cells (Fig. 3e,f) that coexpress inhibitory immunoreceptors, such as Tim-3 (*Havcr2*), Lag-3, CTLA-4 and other molecules characteristic of T cell exhaustion and dysfunction (Fig. 2f). PD-1 expression was significantly higher in Foxp3^–^CD4^+^ T_conv_ cells from *Chat*^fl/fl^; *Cd4-cre* mice than in those from *Chat*^fl/fl^ mice (Fig. 7a,b). Notably, PD-1 levels strongly correlated with HCC grade in *Chat*^fl/fl^; *Cd4-cre* mice but not in *Chat*^fl/fl^ mice (Fig. 7c). We previously reported that loss of ChAT in T cells promotes the expression of PD-1, Tim-3 and Lag-3 during chronic viral infection^27^. In the present study, both CD4^+^ T cells and CD8^+^ T cells in HCC-bearing livers showed a broad trend of upregulation of these inhibitory receptors in the absence of cholinergic T cells (Extended Data Fig. 9a–f). Thus, in the absence of *Chat* in T cells, an unleashing of PD-1 inhibitory activity occurs that may restrict the functions of antitumor T_conv_ cells, allowing HCC progression.

To test this hypothesis, we administered PD-1 blockade antibodies to *Chat*^fl/fl^ and *Chat*^fl/fl^; *Cd4-cre* mice during HCC development (Fig. 7d). As reported for NASH-induced HCC^8^, we did not observe a therapeutic effect of PD-1 blockade in control *Chat*^fl/fl^ mice, perhaps due to ChAT expression by PD-1^+^ T_conv_ cells and the negative effects of cholinergic activity on PD-1 expression. However, PD-1 blockade significantly reduced HCC development in *Chat*^fl/fl^; *Cd4-cre* mice, substantially eliminating the differences in tumor progression (Fig. 7e,f). We did observe two tumor-free animals among anti-PD-1-treated *Chat*^fl/fl^; *Cd4-cre* mice (Fig. 7f), a status rarely seen for this genotype. These data suggest that ChAT invigorates dysfunctional T cells in HCC.

### Cholinergic modulation of TCR-induced Ca^2+^–NFAT signaling

**Fig. 8:**
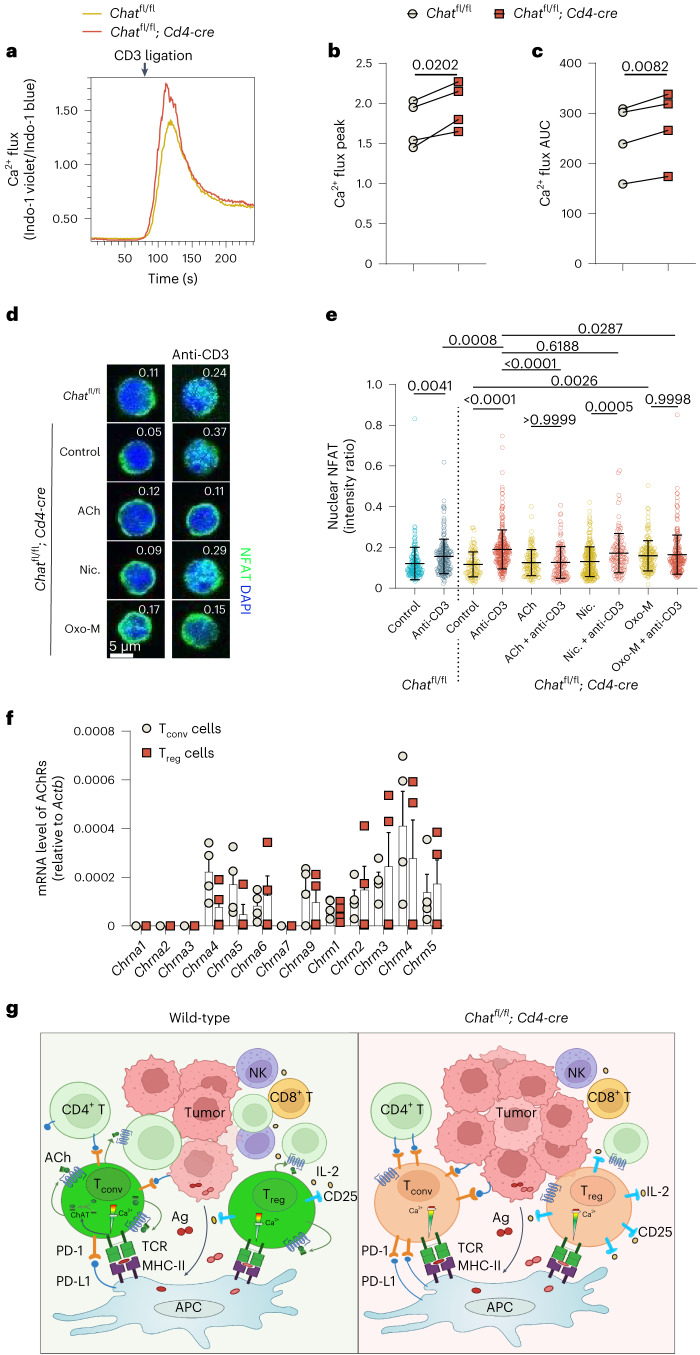
TCR-induced Ca^2+^–NFAT signaling is restrained by cholinergic activity in T cells. **a**–**c**, Representative overlaid kinetics plots of flow cytometry curves (**a**), quantification of Ca^2+^ influx peaks (**b**) and ‘area under curve’ (AUC; **c**) for the Ca^2+^ flux occurring in *Chat*^fl/fl^ and *Chat*^fl/fl^; *Cd4-cre* CD4^+^ T cells. In **b** and **c**, each dot represents T cells from an individual mouse. *Chat*^fl/fl^ and *Chat*^fl/fl^; *Cd4-cre* littermates of both sexes across various ages were paired for analysis, with at least two measurements taken per mouse. *P* values were determined by paired, two-tailed *t*-test; data are a summary of two independent experiments. **d**,**e**, Representative fluorescence micrographs (**d**) and quantification (**e**) of NFAT immunofluorescent staining of splenic CD4^+^ T cells purified from *Chat*^fl/fl^ and *Chat*^fl/fl^; *Cd4-cre* mice. Ratios of nuclear NFAT to cytoplasmic NFAT are displayed in each image in **d** and are statistically compared in **e**. Each dot in **e** represents one cell (*n* = 160 control *Chat*^fl/fl^ T cells; *n* = 180 anti-CD3-treated *Chat*^fl/fl^ T cells; *n* = 101 control *Chat*^fl/fl^; *Cd4-cre* T cells; and *n* = 269, 128, 119, 246, 114, 157 and 212 *Chat*^fl/fl^; *Cd4-cre* T cells treated with anti-CD3, ACh, ACh + anti-CD3, nicotine (Nic), nicotine + anti-CD3, Oxo-M or Oxo-M + anti-CD3). Data are shown as mean ± s.e.m. *P* values were determined by one-way ANOVA with Tukey’s multiple comparisons test and are representative of two independent experiments. **f**, qPCR determination of mRNA levels (relative to *Actb*) of the indicated nAChR (*Chrna1*–*Chrna9*) and mAChR (*Chrm1*–*Chrm5*) genes in T_conv_ and T_reg_ CD4^+^ T cells sorted from livers of HCC-bearing *Foxp3-YFP* mice (*n* = 4); data are representative of three independent experiments. **g**, Diagram summarizing our proposed model of ChAT function in T cells during HCC. In wild-type mice, HCC antigens induce the expression of ChAT in T cells. Autocrine/paracrine cholinergic signaling by ChAT-expressing T cells influences T cell Ca^2+^ homeostasis and regulates TCR-induced Ca^2+^ signaling. Without such cholinergic modulation (as occurs in *Chat*^fl/fl^; *Cd4-cre* mice), TCR-induced Ca^2+^ signaling is hyperactivated, leading to T cell exhaustion, overexpression of PD-1 in T_conv_ cells and increased CD25 in T_reg_ cells. The inhibitory activity of PD-1 is unleashed, and T_reg_-mediated suppression is enhanced, compromising antitumor responses mounted by NK cells and T_conv_ cells. In the absence of ChAT, HCC progression proceeds unabated; Ag, antigen; MHC-II, major histocompatibility complex class II. Source data

CD25 expression is controlled by the transcription factor NFAT, which is regulated by Ca^2+^ signaling^42^. TCR-induced Ca^2+^–NFAT signaling is indispensable for T cell exhaustion and induces the expression of inhibitory surface receptors, such as PD-1, Lag-3 and Tim-3 (ref. ^43^). NFAT-induced transcription factors, including NR4A and TOX, drive T cell exhaustion/dysfunction^44–47^. We therefore sought to determine if cholinergic activity affects Ca^2+^ signaling in T cells. Ca^2+^ influx elicited by TCR engagement was significantly stronger in *Chat*^fl/fl^; *Cd4-cre* T cells than in their *Chat*^fl/fl^ counterparts (Fig. 8a–c). Furthermore, TCR activation-induced nuclear translocation of NFAT was higher in *Chat*^fl/fl^; *Cd4-cre* T cells than in *Chat*^fl/fl^ T cells (Fig. 8d,e). Interestingly, when *Chat*^fl/fl^; *Cd4-cre* T cells were prestimulated with ACh, NFAT nuclear translocation was no longer inducible by TCR activation (Fig. 8d,e). Thus, cholinergic activity in T cells constrains TCR-induced Ca^2+^–NFAT signaling.

ACh regulates intracellular Ca^2+^ levels by binding to nAChRs and mAChRs^48^. We found that T_reg_ cells and T_conv_ cells in livers of HCC-bearing mice expressed similar arrays of AChRs (Fig. 8f). We then applied either a nicotinic agonist or a muscarinic agonist to *Chat*^fl/fl^; *Cd4-cre* T cells in vitro. Nicotine, which activates nAChRs, had no detectable influence on TCR-induced NFAT translocation. By contrast, oxotremorine methiodide (Oxo-M), an mAChR agonist, by itself induced an increase in NFAT nuclear translocation and abolished subsequent TCR-induced NFAT translocation (Fig. 8d,e).

The G_q/11_-coupled M1, M3 and M5 mAChRs elicit Ca^2+^ release from the endoplasmic reticulum (ER) by activating the downstream phospholipase C/inositol 1,4,5-trisphosphate/inositol 1,4,5-trisphosphate receptor (PLC/IP_3_/IP_3_R) cascade^49^, the same pathway triggered by TCR signaling. Mobilization of Ca^2+^ in T cells is a biphasic event divided into the initial releasing of intracellular ER Ca^2+^ stores and the subsequent extracellular Ca^2+^ influx from ‘Ca^2+^ release-activated Ca^2+^’ (CRAC) channels^50^. The CRAC channels can also be triggered by mAChR activation in T cells^51,52^. However, the activation of mAChR depletes the IP_3_-sensitive Ca^2+^ pool (ER Ca^2+^) and renders the T cells unresponsive to further CRAC channel-dependent stimulation^52^. In line with these findings, our results indicate that cholinergic signaling via muscarinic receptors desensitizes T cells to TCR-induced Ca^2+^–NFAT signaling.

### Parallels to human HCC

**Extended Data Fig. 10:**
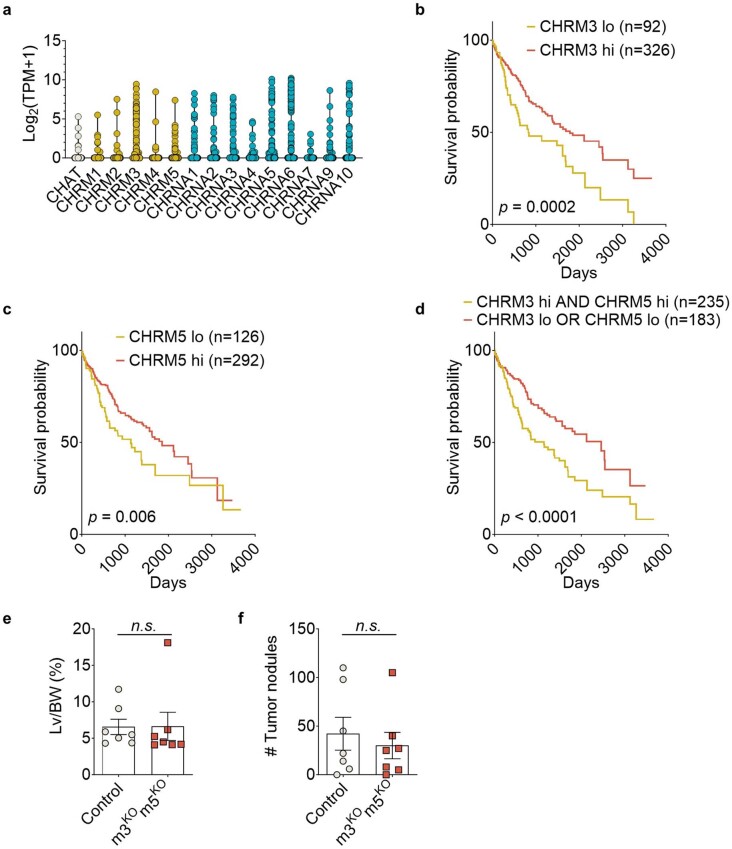
Cholinergic signaling is associated with human HCC. **a** Violin plots showing the expression of *CHAT* and the indicated muscarinic acetylcholine receptors and nicotinic acetylcholine receptors (alpha subunits) in T cells isolated from patient HCC samples (GSE98638). **b-d** Survival of HCC patients with high expression of *CHRM3* (b), *CHRM5* (c), or both *CHRM3* and *CHRM5* (d). Data are from the TCGA database. **e,f** Ratios of liver weight to body weight (e) and numbers of tumor nodules (f) of *Chat*^*fl/fl*^ and *Chat*^*fl/fl*^*;CD4-Cre* mice on day 20 of HCC induction with standard vectors or plus vectors carrying gRNAs for Chrm3 and Chrm5. Each dot represents an individual mouse (n=7 mice per group). Data are the mean ± SEM. *n.s*., not significant, determined by unpaired, two-tailed t-test. Source data

We examined an scRNA-seq dataset^34^ and noted that T cells from individuals with HCC expressed *CHAT* and an array of mAChRs and nAChRs (Extended Data Fig. 10a). To further study this association between cholinergic signaling and human HCC, we examined HCC cases profiled by The Cancer Genome Atlas (TCGA). High expression of *CHRM3* or *CHRM5* in samples from individuals with HCC was positively correlated with a favorable prognosis (Extended Data Fig. 10b–d). These observations prompted us to use our mouse model to determine if *Chrm3* and *Chrm5* expressed by HCC cells were acting to directly suppress HCC development. To this end, we designed CRISPR vectors to target M3 and M5 mAChRs and induced *Chrm3*/*Chrm5*-knockout HCC. We found that the knockout of M3 and M5 AChRs in mouse HCC cells did not significantly affect HCC development (Extended Data Fig. 10e,f). Therefore, our data do not support the direct suppression of HCC by CHRM3 and CHRM5 in HCC cells. We do acknowledge that there could be compensation by other receptors or interspecies differences.

Collectively, our study supports a model (Fig. 8g) whereby tumor antigens induce the expansion of ChAT-expressing T_reg_ cells and PD-1^+^ T_conv_ cells. ACh produced by these T cells modulates TCR-induced Ca^2+^ signaling to prevent its hyperactivity. In the absence of such cholinergic modulation, the Ca^2+^–NFAT pathway becomes hyperactivated to increase immunosuppression by T_reg_ cells and to impose dysfunction on T_conv_ cells, resulting in compromised antitumor immunity.

## Discussion

The immune milieu of the liver at steady state favors tolerance over immune responses to prevent overreaction to innocuous antigens from food, microbial substances or by-products of metabolism. Liver T_reg_ cells have a crucial function in the maintenance of this peripheral tolerance state^53^. In the livers of individuals with HCC, T_reg_ cell infiltration is prominently elevated, alongside the exhaustion and impaired function of CD8^+^ T cells^34,54^. The clonal expansion of T_reg_ cells and exhausted T cells in individuals with HCC has been revealed by scRNA-seq^34^. Using scRNA-seq and flow cytometric analysis, we have demonstrated the induction of Foxp3^+^ T_reg_ cells and PD-1^+^ T_conv_ cells in our mouse HCC model and have shown that ChAT-expressing T cells predominantly belong to these two populations.

The clonal expansion of ChAT-expressing T_reg_ cells and PD-1^+^ T_conv_ cells in our model appears to be driven by tumor antigens. It should be stressed that our HCC model does not involve viral vectors or mutant proteins; as such, the tumor antigens involved here are likely ‘self’ proteins. Such proteins are postulated to be weakly immunogenic, particularly within the tolerogenic immune milieu in the liver. Indeed, the T cell immune response to HCC antigens is reportedly dysfunctional and unreactive to HCC^55^. Our data suggest that ChAT deficiency in T cells exacerbates the dysfunction of T_conv_ cells and reinforces the immunosuppressive function of T_reg_ cells, significantly compromising immunosurveillance against liver cancer. We used OVA to mimic a ‘foreign’ tumor antigen by inducing OVA HCC in immunized mice. We expected that HCC onset in this situation would induce a potent antitumor immune response rather than the accumulation of T_reg_ cells and dysfunctional T_conv_ cells. Indeed, OVA HCC development was prevented to the same degree in *Chat*^fl/fl^ and *Chat*^fl/fl^; *Cd4-cre* mice. Thus, the cholinergic activity of T cells is most relevant when triggered by ‘self’ tumor antigens.

Many actions of ACh in the nervous system are mediated through Ca^2+^ signaling via either G-protein-coupled mAChRs or ionotropic nAChRs^48^. nAChRs are ACh-gated ionic channels with a variable range of permeability to Ca^2+^. The M1, M3 and M5 mAChRs are coupled with G_q/11_ and elicit the PLC/IP_3_/IP_3_R cascade to trigger ER Ca^2+^ release, whereas M2 and M4 mAChRs are generally linked via G_i/o_ to cAMP production^48^. We demonstrated that these three classes of AChRs are expressed by both T_conv_ cells and T_reg_ cells in HCC-bearing livers. In T lymphocytes, M1 mAChR activation and TCR engagement rely on the same molecular pathway to trigger Ca^2+^ influx. Due to this overlap, Ca^2+^ signaling events triggered by these pathways are mutually exclusive^52^. In the Premack study, the maximal cholinergic stimulation mediated by overexpressed M1 receptor completely emptied the ER Ca^2+^ store, the Ca^2+^ pool essential for TCR-induced Ca^2+^ signaling. Moreover, the ER Ca^2+^ release mediated by cholinergic signaling occurred in a quantal manner: a submaximal concentration of cholinergic agonist rapidly released a fraction of the ER Ca^2+^ store, followed by a slower or terminated Ca^2+^ release^56,57^. Certain mechanisms, including the inactivation of IP_3_ receptors, can be triggered to attenuate ER Ca^2+^ release^57,58^. During persistent submaximal cholinergic stimulation, the ER Ca^2+^ release elicited by other IP_3_-dependent agonists is dampened^56^. Due to the modest expression levels of AChRs by T cells and the ubiquitous presence of acetylcholinesterase^59^, we speculate that autocrine/paracrine ACh signaling by T cells might operate in such a submaximal way. Accordingly, although we did not observe significant differences in basal levels of Ca^2+^ influx and NFAT nuclear translocation between *Chat*^fl/fl^ and *Chat*^fl/fl^; *Cd4-cre* T cells, both TCR-induced Ca^2+^ influx and NFAT nuclear translocation were elevated in the absence of cholinergic activity in T cells. Moreover, ACh pretreatment inhibited TCR-induced NFAT nuclear translocation. Collectively, these data allow us to propose a model in which autocrine/paracrine ACh produced by ChAT-expressing T cells affects their Ca^2+^ homeostasis. This altered Ca^2+^ status restrains the expression of CD25 by T_reg_ cells and the expression of PD-1 and other exhaustion markers by T_conv_ cells. In the absence of ChAT in T cells, hyperimmunosuppressive T_reg_ cells and dysfunctional T_conv_ cells interfere with adaptive and innate antitumor responses and permit HCC progression.

In conclusion, our study shows that lymphocytes are the dominant cholinergic cells in HCC-bearing livers and that ChAT-expressing T cells orchestrate immune responses against HCC. Our results may prompt investigation of how lymphocyte-mediated cholinergic regulation of liver carcinogenesis can be exploited to enhance antitumor immune responses in individuals with liver cancer.

## Methods

### Mice

*Chat-GFP* (B6.Cg-Tg(RP23-268L19-EGFP)2Mik/J), *Chat*^fl^ (B6.129-*Chat*^*tm1Jrs*^/J), *Cd4-cre* (Tg(*Cd4-cre*)1Cwi/BfluJ), *Il21r*^–/–^ (B6.129-*Il21r*^*tm1Kopf*^/J), OT-II (B6.Cg-Tg(*TcraTcrb*)425Cbn/J), Confetti (*Gt*(*ROSA*)*26Sor*^*tm1*(*CAG-Brainbow2.1*)*Cle*^/J), *Foxp3*^YFP/Cre^ (B6.129(Cg)-*Foxp3*^*tm4*(*YFP/icre*)*Ayr*^/J), NSG (NOD.Cg-*Prkdc*^*scid*^ *Il2rg*^*tm1Wjl*^/SzJ) and control NOD/ShiLtJ mice were all purchased from the Jackson Laboratory and bred in the animal facility at the Princess Margaret Cancer Centre. The mice were housed on ventilated racks supplied with autoclaved microisolator cages. Reverse osmosis water was supplied through an automatic watering system. The light cycle was lights off at 1800 h and lights on at 0600 h. The ambient temperature was held between 22 and 23 °C with a humidity of 40–60%. Mice were routinely fed on the irradiated 7012 Teklad LM-485 Mouse/Rat Sterilizable Diet. All animal experiments were approved by the University Health Network Animal Care Committee.

### CRISPR and transposon vectors

Single guide RNAs (sgRNAs) targeting *Trp53* and *Pten* were as previously described^21^. The guide oligonucleotides were designed to express mouse *Pten* sgRNA and *Trp53* sgRNA (Supplementary Table 1). The annealed double-stranded guide oligonucleotides were cloned into the BbsI cut sites of the pX330 vector^60^. The *Trp53* sgRNA cassette in pX330-p53 was amplified by PCR with primers (Supplementary Table 1). This additional sgRNA cassette was then cut with NheI and XbaI and subcloned into the NheI site of pX330-Pten to obtain the duplex CRISPR vector pX330-p53-Pten.

The transposon system using SB100X and pT2/BH was as described previously^61^. The mouse *Myc* coding sequence was cloned into pT2/BH with EcoR1 and NotI restriction enzymes to obtain the pT2-Myc plasmid.

A modified puromycin-T2A-NLS-Cre version of the pX330 vector was generated. In brief, this vector contained an additional expression cassette under the control of the mouse PGK promoter (driving expression of the puromycin-resistance gene), a T2A self-cleaving peptide flanked by flexible GSG linkers and a P1 bacteriophage Cre recombinase engineered with an N-terminal nuclear localization site and an HSVpA signal. This vector was further modified to express both mouse *Pten* and *Trp53* U6 promoter-driven gRNAs following the process used to build pX330-p53-Pten (see above).

For the pT2-OVA-P2A-Myc plasmid, a 660-base pair (bp) fragment of the cytosolic region (amino acids 173–386, containing both major histocompatibility class I- and II-restricted epitopes) of OVA cDNA was amplified by PCR and engineered with a Kozak consensus methionine and EcoRI and NheI cloning sites using the following primers: OVA_ERI_U1 and OVA_Nhe1_L1 (Supplementary Table 1). A P2A self-cleaving 2A peptide cassette, flanked by a flexible GSG linker region, was then added in-frame to the 3′ end of the OVA region using NheI and XhoI restriction sites. Finally, the mouse *Myc* cDNA was subcloned in-frame 3′ of the P2A cassette using PCR primers Cmyc_XhoI_LE_U1 and Cmyc_BstB1_L1 (Supplementary Table 1). This primer set removed the mouse c-Myc start methionine and engineered an additional BstBI site at the 3′ end of mouse *Myc* that allowed subcloning of the entire Kozak OVA-P2A-Myc cassette into the pT2/BH-CAG-GS-Myc plasmid using EcoRI and BstBI restriction enzyme cloning sites.

For the pT2-EF1a-rtTA-P2A-Myc+TRE-OVA plasmid, the entire ~1,700-bp CAG-CMV promoter and enhancer region in the pT2-OVA-P2A-Myc plasmid was removed and replaced with the 289-bp human elongation factor 1-α (EF1a) core promoter to create the pT2-EF1a-OVA-P2A*-*Myc plasmid. The pT2-EF1a-OVA-P2A*-*Myc plasmid was then digested with EcoRI and NheI to remove the chicken OVA region and replace it with a reverse tetracycline-controlled transactivator (rtTA) coding sequence (rtTA-advanced) to create the pT2-EF1a-rtTA-P2A*-*Myc plasmid. TRE tight Promoter from pTRE-Tight caspase-3 (p12)::nz [TU#817] (a gift from M. Chalfie (Columbia University), Addgene plasmid 16084) was subcloned upstream of the BGH 3′ untranslated region and poly(A) signal of pcDNA3.1-Zeo (Invitrogen) via XhoI and EcoRI restriction sites to generate pcDNA3.1(–)Zeo-TRE-BGHpA. Next, OVA cDNA was subcloned into pcDNA3.1(–)Zeo-TRE-BGHpA using EcoRI and HindIII restriction sites to generate pcDNA3.1(–)Zeo-TRE-OVA-BGHpA. The entire 1,256-bp TRE-OVA-BGHpA fragment was PCR amplified using primers BGHpA_IF_Fwd and TRE_IF_Rev (Supplementary Table 1). The amplified fragment was then cloned in the reverse ‘*trans*’ orientation into HindIII-linearized pT2-EF1a-rtTA-P2A*-*Myc plasmid using In-Fusion (Takara Bio), generating the final pT2-EF1a-rtTA-P2A-Myc+TRE-OVA plasmid.

To induce *Chrm3*/*Chrm5*-knockout HCC, the guide oligonucleotides were designed to express mouse *Chrm3* sgRNA and *Chrm5* sgRNA (Supplementary Table 1). The annealed double-stranded guide oligonucleotides were cloned into the BbsI cut sites of a modified version of the pX330 vector from which the Cas9 cassette had been removed. The *Chrm3* and *Chrm5* sgRNA expression vectors were combined with pX330-p53-Pten, SB100X and pT2-Myc for HCC induction; in this way, the *Chrm3* and *Chrm5* sgRNAs only targeted the cells receiving the pX330-p53-Pten vector.

### Hydrodynamic injections to induce HCC

For delivery of transposon and CRISPR vectors, mice (8–20 weeks old) were injected with a volume of 100 ml per kg (body weight) containing 25 μg of pX330-p53-Pten (or its modified form) plus 0.66 μg of SB100X and 5 μg of pT2-Myc (or its modified forms). The molar ratio of SB100X to pT2-Myc was 1 to 5. Hydrodynamic injection into the lateral tail vein took 5–7 s. Blinding was achieved during injection by putting littermates of different genotypes into new cages lacking mouse information.

To evaluate and quantify HCC development, mice were monitored daily for the appearance of palpable tumors, which were defined as a discernable enlargement of the abdomen. The humane endpoint was reached when one of the following criteria was met: a 10% increase in body weight from baseline, an estimated liver weight of over 5 g determined by the degree of abdominal distension, moribund condition or persistent facial displays of pain and distress. For the survival curve, the exact dates when mice reached the humane endpoint were determined after killing mice bearing significant HCC. Livers collected at this stage were 3–7 g in weight, and the exact endpoints (liver weight over 5 g) were adjusted by 1 d per g (live weight). Early mortalities (<5 d) were considered to be due to injection-associated death and were removed from the analysis. The number of liver tumors was quantified by counting tumor nodules on the surface of the liver or by normalizing the number of tumor clones to the area of liver sections. Blinding was performed in quantification of liver sections but not surface tumor nodules.

### Antibodies and treatments

Antibodies used to deplete mice of NK cells, CD4^+^ T cells, CD8^+^ T cells or CD25^+^ T_reg_ cells or antibodies used for PD-1 blockade (and isotype control antibodies) were from BioXCell. Briefly, anti-NK1.1 (clone PK136) and anti-CD4 (clone GK1.5) were injected intraperitoneally (i.p.) at 100 μg per mouse on days −1, 2, 7, 12 and 16 of standard HCC induction. Anti-CD8 (clone 2.43) was injected i.p. at 100 μg per mouse on days −1, 2, 7, 12, 16 and 21 of standard HCC induction. Anti-CD25 (clone PC-61.5.3) was injected i.p. at 150 μg per mouse on days 1, 6, 11 and 17 of standard HCC induction. To analyze the efficiency of T_reg_ cell depletion, *Foxp3-YFP* mice were injected at the same time, and anti-mouse CD25–Alexa Fluor 647 (clone 7D4, BD) was used for flow cytometry. Anti-PD-1 (clone RMP1-14) was injected i.p. at 100 μg per mouse on days 3, 7, 11 and 15 of standard HCC induction.

To induce NASH-derived HCC, male mice (4–6 weeks old) were fed a Western diet (Research Diets, D16022301i) for 15 months. The diet contained 40 kcal% fat, 20 kcal% fructose and 2% cholesterol.

For OVA immunization, mice were injected into the base of their tails with 50 μl of OVA/complete Freund’s adjuvant emulsion (Hooke Laboratories, EK-0301).

To induce OVA expression in HCC, HCC was induced in mice by injection of pT2-EF1a-rtTA-P2A-Myc+TRE-OVA. When mice showed a discernable enlargement of the abdomen, they received Dox-containing drinking water (600 mg liter^–1^; Sigma) for 15 d, followed by euthanasia and fluorescence-activated cell sorting analysis of liver cells isolated as described below.

### Hepatic MNC isolation

Mice were killed by CO_2_ asphyxiation and immediately subjected to whole-body perfusion with ice-cold PBS containing 10 mM EDTA. Liver tissues were collected, disrupted and passed through 70-μm sieves to obtain single-cell suspensions. MNCs were enriched by centrifugation through a 40%/80% Percoll gradient for 20 min at 2,000 r.p.m.

### Flow cytometry

Antibodies included anti-mouse CD4–BUV737 (612843), anti-mouse CD8–PerCP-Cy5.5 (551162), anti-mouse CD19–BUV395 (563557), anti-mouse CD25–Alexa Fluor 647 (clone 7D4, 563598) and anti-mouse CD62L–BUV737 (612833) from BD; anti-mouse CD45.2–Alexa Fluor 700 (109822), anti-mouse NK1.1–BV605 (108740), anti-mouse CD11b–BV510 (101263), anti-mouse CD44–Alexa Fluor 700 (103026), anti-mouse OX40–PE (119409), anti-mouse CD4–BV510 (100559), anti-mouse CD45–PerCP-Cy5.5 (103132), TCR Vβ5.1/Vβ5.2–PE-Cy7 (139508), anti-mouse CD25–PE-Cy7 (clone PC-61, 102016), anti-mouse CD4–PE (100408), anti-mouse CD62L–FITC (104406), anti-mouse CD44–APC (103012), anti-mouse CD8–APC-Cy7 (100714), anti-mouse PD-1–APC (109112), anti-mouse Tim-3–PE (119704), anti-mouse Lag-3–PerCP-Cy5.5 (125219), anti-mouse PD-L1–PE-Cy7 (124314), anti-mouse CTLA-4–PE (106306), anti-mouse IFNγ–APC (505810) and anti-mouse IL-17A–BV605 (506927) from BioLegend and anti-mouse FOXP3–PE (clone FJK-16S, 12-5773-82) from Thermo Fisher. These antibodies were used at a 1:200 dilution. Mouse CD1d–PBS-57 BV421-labeled tetramer was from the NIH Tetramer Facility. We determined the appropriate concentration of the CD1d tetramer by conducting a pilot experiment on each lot, and either a 1:1,000 or 1:400 dilution was used.

For intracellular cytokine staining, MNCs were stimulated for 4 h with 1 mg ml^–1^ ionomycin plus 25 μg ml^–1^ phorbol myristate acetate in the presence of BD GolgiPlug. Cytokine staining was performed with Cytofix/Cytoperm kits (BD) following the manufacturer’s instructions. For staining of transcription factors, the eBioscience Foxp3/Transcription Factor Staining Buffer Set was used following the manufacturer’s instructions. Anti-GFP Alexa Fluor 488 (Thermo Fisher, A21311) were used to label GFP in intracellular staining analyses.

Flow cytometric analyses were performed using BD LSRFortessa cell analyzers at the Princess Margaret Flow Facility.

### scRNA-seq and data analysis

HCC was induced in Chat–GFP mice using pX330-p53-Pten plus SB100X and pT2-Myc. HCC livers (from two male and two female mice) and control livers (from sex-matched littermates) were collected for hepatic MNC isolation on day 26 of HCC induction. Hepatic MNCs were stained with antibodies to CD4, CD8, CD19, TCRβ, NK1.1, CD45 and CD1d tetramer as well as with barcode antibodies (TotalSeq-C0304, C0305, C0306 or C0307) to hashtag cells from individual mice of the HCC or control group. CD45^+^DAPI^–^NK1.1^–^CD1dTetramer^–^CD19^–^TCRβ^+^CD4^+^CD8^–^GFP^+^ (ChAT–GFP^+^CD4^+^ T cells) and CD45^+^DAPI^–^NK1.1^–^CD1dTetramer^–^CD19^−^TCRβ^+^CD4^+^CD8^–^GFP^–^(ChAT–GFP^–^CD4^+^ T cells) populations were sorted on a FACSAria Fusion cell sorter (BD) at the Princess Margaret Flow Facility, with the two CD4^+^ T cell compartments individually sorted from each mouse. The same CD4^+^ T cell compartments from mice receiving the same treatment were pooled into four samples: control ChAT–GFP^+^, control ChAT–GFP^–^, HCC ChAT–GFP^+^ and HCC ChAT–GFP^–^. After sorting and pooling, the samples were immediately submitted to the Princess Margaret Genomics Centre for downstream processing. The four samples were loaded onto a 10x Chromium Controller, and libraries were prepared using a Chromium Next GEM Single Cell 5′ HT Reagent kit v2 (Dual Index, 10x Genomics). The libraries were sequenced on an Illumina NovaSeq 6000 instrument. The sequencing depths were GEX ~50,000 reads per cell, TCR ~5,000 reads per cell and cell hashing TotalSeq C ~2,000 reads per cell.

For scRNA-seq data analysis, sequencing data were processed using Cell Ranger (version 7.0.0) and aligned to the annotated mouse genome (mm10). The Cell Ranger VDJ pipeline was used to call TCR sequences. The clonotype analysis was performed on the merged contig annotations of four samples. The junctions of the *V*, *D* and *J* segments were determined with the IMGT database. The filtered feature barcode matrices in the hierarchical data format (.h5 files) and .csv files of filtered contig annotations from the four samples were analyzed with Partek Flow software (version 10.0.23.0214, license from the Centre for PanorOmic Sciences Bioinformatics Core) and analyzed together. With the ‘split by feature type’ tool, the single-cell counts were split into two data nodes: gene expression and antibody capture. After excluding low-quality cells (counts of <30,000; percentage of mitochondrial counts of <30), gene expression was normalized using the recommended counts per million method. Antibody capture was normalized with the recommended method (add 1.0, divide by geometric mean, add 1.0 and log 2.0), and the multiplets and cells with ambiguous hashing were filtered out. These two sets of data were then merged with the ‘merge matrices’ tool to obtain the filtered, uniquely hashtagged and normalized counts (15,703 cells in total). This counts data node was resplit to generate new gene expression and antibody capture nodes. Dimensionality reduction and visualization were performed on the new gene expression data node using ‘PCA’ (number of principal components: 100; features contribute: by variance; split by sample: no), ‘graph-based clusters’ (with default parameters except the resolution was set to 1.0) and ‘UMAP’ (with default parameters) tools. ‘Compute biomarkers’ was performed on graph-based clustering results to identify marker genes of each cell cluster. Differential analyses were performed using ‘GSA’ and visualized using heat maps and volcano plots.

### Immunohistological analyses

Sections cut from formalin-fixed, paraffin-embedded blocks of mouse livers were used for immunohistochemistry (IHC). After dewaxing and rehydration, endogenous peroxidase was deactivated in 3% hydrogen peroxide (20 ml of 30% hydrogen peroxide + 180 ml of PBS) for 15 min at room temperature. Antigen retrieval with 10 mM sodium citrate buffer (pH 6.0) was performed before immunostaining.

Primary antibodies used for IHC included goat anti-GFP (Novus, NB100-1678), rat anti-Foxp3 (Thermo Fisher, clone FJK-16s), rabbit anti-CD3 (Abcam, ab5690), rabbit anti-CD11b (Abcam, ab133357), rabbit anti-p53 (Vector Labs, VP-P956), rabbit anti-c-MYC (Cell Signaling, 5605) and rabbit anti-PTEN (Cell Signaling, 9559). Polymer-conjugated secondary antibodies included alkaline phosphatase (AP) goat anti-rat IgG (MP-544415), horseradish peroxidase (HRP) goat anti-rat IgG (MP-5444), HRP horse anti-goat IgG (MP-7405), HRP horse anti-rabbit IgG (MP-7405) and AP horse anti-rabbit IgG (MP-5401; all from Vector Laboratories). ImmPACT Vector Red Substrate kits, including AP substrate (SK-5105) and DAB peroxidase substrate (SK-4100; both from Vector Laboratories), were used for chromogenic detection.

Immunostained histological sections were scanned using a NanoZoomer 2.0-HT slide scanner from Hamamatsu. Quantification of immune cell clusters in scans of H&E-stained liver sections and determination of c-MYC, p53 and PTEN expression in tumor clones present in scans of IHC-stained liver sections were performed using NDP.view2 (Hamamatsu) in a blinded fashion. For analysis of c-MYC expression in liver sections, random 20× non-tumor fields of the c-MYC IHC scans from liver sections of individual mice were output using NDP.view2 and blindly analyzed using ImageJ (https://imagej.nih.gov/ij/) with the plugin ‘IHC Profiler’^62^.

For the immunofluorescence staining shown in Extended Data Fig. 1f, Alexa Fluor 568-conjugated goat anti-rabbit IgG (Thermo Fisher, A-11036) was used.

For detection of fluorescent proteins in livers of *Rosa26*^Confetti^ mice, frozen liver sections were fixed with 2% paraformaldehyde for 8 min at room temperature and directly observed using an Olympus FluoView FV1000 confocal laser-scanning microscope.

### Measurement of Ca^2+^ flux

CD4^+^ T cells were purified from spleens of *Chat*^fl/fl^ and *Chat*^fl/fl^; *Cd4*-*cre* mice using a CD4^+^ T Cell Isolation kit (Miltenyi Biotec) and autoMACS following the manufacturer’s instructions. Purified CD4^+^ T cells were loaded with 1 μM Indo-1 Ca^2+^ indicator (Thermo Fisher) in a 37 °C water bath for 45 min. After washing, CD4^+^ T cells were stained with anti-CD4–PE, anti-CD62L–FITC and anti-CD44–APC at 4 °C for 30 min and stained with 1 μg ml^–1^ hamster anti-CD3 (Biolegend, 100359, clone 145-2C11) at 4 °C for 30 min. To activate TCR signaling, rabbit anti-hamster (Jackson ImmunoResearch, 307-005-003) was added at 10 μg ml^–1^ at the indicated time during flow cytometric analysis. CD4^+^CD62L^+^CD44^–^ naive CD4^+^ T cells were gated for comparison.

### Analysis of NFAT nuclear translocation

CD4^+^ T cells were purified from spleens of *Chat*^fl/fl^ and *Chat*^fl/fl^; *Cd4*-*cre* mice using a CD4^+^ T Cell Isolation kit (Miltenyi Biotec) and autoMACS following the manufacturer’s instructions. Purified CD4^+^ T cells were resuspended in serum-free RPMI medium at 5 × 10^5^ cells per ml. These CD4^+^ T cell suspensions were incubated with ACh (Sigma), nicotine (Tocris Bioscience) or Oxo-M (Tocris Bioscience) at 37 °C for 15 min. Anti-CD3/CD28 microbeads (Thermo Fisher) were then added to the treated T cell suspensions at a 1:1 bead:cell ratio, and the cells were cultured for another 15 min. After removing the anti-CD3/CD28 microbeads with a magnetic block, CD4^+^ T cells were fixed and prepared for cytospins with a cytocentrifuge (Thermo Fisher). The cytospins were permeabilized with 1% Triton X-100 for 30 min, blocked with 3% fetal bovine serum, 1% bovine serum albumin and 0.3% Triton X-100 in PBS for 30 min and stained with Alexa Fluor 488-conjugated anti-NFAT1 diluted 1:50 (clone D43B1, Cell Signaling) overnight at 4 °C. After washing and counterstaining with DAPI, the cytospins were mounted with cover slides and examined with a Nikon A1R confocal microscope. Fluorescence micrograms were acquired using a ×40 objective lens. Consecutive images across the diameter of each cytospin (about 16 shots) were collected and quantified. CellProfiler (v4.2.1) was used for the quantification of NFAT nuclear translocation. The minimum cross-entropy thresholding method was used to identify the objects. The MeasureObjectIntensityDistribution module was used for the analysis of NFAT distribution. Briefly, the radial distribution of NFAT staining was measured with three concentric rings (bins) starting from the center of DAPI staining. The ratio of the fraction of total NFAT intensity in the first bin (innermost ring) versus the third bin (outermost ring) was designated as the parameter for NFAT nuclear translocation. The CellProfiler project file will be shared upon inquiry.

### Analysis of human HCC datasets

Expression levels of *CHAT* in various clusters of T cells isolated from tumor tissue and adjacent liver tissue of individuals with HCC were determined by examining the scRNA-seq dataset of immune cells of human HCC previously published by Zhang et al.^32^. Violin plots were generated using their interactive web-based tool (http://cancer-pku.cn:3838/HCC/). Expression levels of *CHAT*, mAChRs and nAChRs (α-subunits) in T cells isolated from samples from individuals with HCC were determined by extracting data from a published scRNA-seq dataset (GSE98638) on T cells from individuals with HCC^34^. Survival curves of individuals with HCC with high expression of *CHRM3* (cutoff was set to log_2_ (fragments per kilobase of transcript per million mapped reads upper quartile (FPKM-UQ) + 1) = 11) and *CHRM5* (cutoff was set to log_2_ (FPKM-UQ + 1) = 9.6) were generated using data obtained from the TCGA Research Network (https://www.cancer.gov/tcga) and analyzed with University of California, Santa Cruz, Xena (http://xena.ucsc.edu/).

### Statistics and reproducibility

Pilot experiments were used to estimate the sample size necessary to generate statistically significant results using the appropriate statistical tests. Genetically modified mice and their littermate controls were used for all experiments where possible. For comparing liver weights and nodule numbers, five to ten mice per group were sufficient to achieve statistical significance. For survival curves, a cohort of 10–20 mice per group was used. To account for potential technical failures, including missed hydrodynamic injection and early mortality associated with injection, we usually included an extra 10% of mice per group. Early mortalities (<5 d) were considered to be due to injection-associated death and were removed from the analysis. The numbers of replicates and independent experiments have been stated in the figure legends. The attempts at replication were successful.

To generate statistically appropriate numbers, it was usually necessary to use more than three litters of mice for each experiment. To control for the treatments (including plasmids and antibodies), mice from each litter were randomly divided into groups to guarantee that sex-matched and genotype-matched individuals obtained different treatments. This grouping was performed ahead of each experiment. For vector delivery by hydrodynamic injection, blinding was achieved during injection by placing littermates of different genotypes into new cages lacking mouse information. Blinding was also performed for quantitative analyses of liver sections.

### Statistical analyses

The Kolmogorov–Smirnov test was used to evaluate normality. Pair-wise comparisons were assessed using two-tailed unpaired Student’s *t*-tests unless otherwise denoted in the figure legends. Data are presented as mean ± s.e.m. unless otherwise indicated. GraphPad Prism 8 and Microsoft Excel (version 2019) were used for the statistical analyses. *P* values of <0.05 were considered statistically significant.

### Reporting summary

Further information on research design is available in the Nature Portfolio Reporting Summary linked to this article.

### Supplementary information

Supplementary Information Supplementary Table 1. Oligonucleotide sequences for sgRNAs and PCR amplifying fragments.

Reporting Summary

### Source data

Source Data Fig. 1 Statistical source data.

Source Data Fig. 2 Statistical source data.

Source Data Fig. 3 Statistical source data.

Source Data Fig. 4 Statistical source data.

Source Data Fig. 5 Statistical source data.

Source Data Fig. 6 Statistical source data.

Source Data Fig. 7 Statistical source data.

Source Data Fig. 8 Statistical source data.

Source Data Extended Data Fig. 1 Statistical source data.

Source Data Extended Data Fig. 2 Statistical source data.

Source Data Extended Data Fig. 3 Statistical source data.

Source Data Extended Data Fig. 6 Statistical source data.

Source Data Extended Data Fig. 7 Statistical source data.

Source Data Extended Data Fig. 8 Statistical source data.

Source Data Extended Data Fig. 9 Statistical source data.

Source Data Extended Data Fig. 10 Statistical source data.

## Data Availability

scRNA-seq data that support the findings of this study have been deposited in the Gene Expression Omnibus under accession code GSE231322. The scRNA-seq datasets of human HCC analyzed in this study include those published by Zheng et al.^34^ and Zhang et al.^32^. The accession code of the Zheng et al. data is GSE98638. The accession codes of the Zhang et al. data are GSE140228 and EGAS00001003449. The human liver HCC (TCGA-LIHC) data analyzed in this study were derived from the TCGA Research Network (http://cancergenome.nih.gov/). All other data supporting the findings of this study are available from the corresponding author upon reasonable request. Source data are provided with this paper.

## Code availability

Details of software versions, plugins, modules and parameters are specified in relevant sections in the Methods. The code for Circos plots showing the distribution of TCR types among GFP^+^ and GFP^–^ T cells from control and HCC-bearing mice has been deposited in a Zenodo repository (https://zenodo.org/record/8058337)^63^.
