# Supplementary material for: Tumor-specific cholinergic CD4+ T lymphocytes guide immunosurveillance of hepatocellular carcinoma
Source: Nat Cancer. 2023 Aug 28;4(10):1437–54. doi: 10.1038/s43018-023-00624-w (PMC10597839; doi:10.1038/s43018-023-00624-w)
Supplement: Supplementary file 1 — Supplementary Table 1. Oligonucleotide sequences for sgRNAs and PCR amplifying fragments. [file 43018_2023_624_MOESM1_ESM.pdf]

nature cancer

Article https://doi.org/10.1038/s43018-023-00624-w

Tumor-specific cholinergic $CD4^+$ T lymphocytes guide immunosurveillance of hepatocellular carcinoma

In the format provided by the authors and unedited

Supplementary Table 1. Oligo sequences for sgRNAs and PCR amplifying fragments

Oligo ID	Oligo sequence
Pten_U1	5’- CACCGCTAACGATCTCTTTGATGA -3’
Pten_L1	5’- AAACTCATCAAAGAGATCGTTAGC -3’
Trp53_U1	5’- CACCGCCTCGAGCTCCCTCTGAGCC -3’
Trp53_L1	5’- AAACGGCTCAGAGGGAGCTCGAGGC -3’
sgTrp53_cassette_Fwd	5’- GCTTCTAGACATGTGAGGGCCTATTTC -3’
sgTrp53_cassette_Rev	5’- TACAGCTAGCGCCATTTGTCTGCAGAATTGG -3’
OVA_ERI_U1	5'- GAATTCGCCGCCATGGTTCTGGTTAATGCCATTGTCTTC -3'
OVA_Nhe1_L1	5'- GCTAGCAGGGGAAACACATCTGCCAAAGAAGAGAAC -3'
Cmyc_XhoI_LE_U1	5'- CTCGAGCCCCTCAACGTGAACTTCACCAAC -3'
Cmyc_BstB1_L1	5'- CAATTAGTTCGAAGTTTATGCACCAGAGTTACGAAGCTGTTCGAGTTTGTGTTTC -3'
BGHpA_IF_Fwd	5’- CCTGCAGCCCAAGCTTGTTCTTTCCGCCTCAGAAGCC -3’
TRE_IF_Rev	5’- AGCCTTCCACAAGCTTCTCGAGTTTACTCCCTATCAGTG -3’
Chrm3_U1	5’- CACCGACCAAGACATTGCCGACAA -3’
Chrm3_L1	5’- AAACTTGTCGGCAATGTCTTGGTC -3’
Chrm5_U1	5’- CACCGCGCCGTGCCGAAGGTGATGG -3’
Chrm5_L1	5’- AAACCCATCACCTTCGGCACGGCGC -3’
