# Supplementary material for: Tumor-specific cholinergic CD4+ T lymphocytes guide immunosurveillance of hepatocellular carcinoma
Source: Nat Cancer. 2023 Aug 28;4(10):1437–54. doi: 10.1038/s43018-023-00624-w (PMC10597839; doi:10.1038/s43018-023-00624-w)
Supplement: Supplementary file 2 — Reporting Summary [file 43018_2023_624_MOESM2_ESM.pdf]

nature portfolio

Corresponding author(s): Tak W. Mak

Last updated by author(s): Jul 22, 2023

Reporting Summary

Nature Portfolio wishes to improve the reproducibility of the work that we publish. This form provides structure for consistency and transparency in reporting. For further information on Nature Portfolio policies, see our Editorial Policies and the Editorial Policy Checklist.

Statistics

For all statistical analyses, confirm that the following items are present in the figure legend, table legend, main text, or Methods section.

n/a	Confirmed	
☐	☒	The exact sample size (*n*) for each experimental group/condition, given as a discrete number and unit of measurement
☐	☒	A statement on whether measurements were taken from distinct samples or whether the same sample was measured repeatedly
☐	☒	The statistical test(s) used AND whether they are one- or two-sided<br>*Only common tests should be described solely by name; describe more complex techniques in the Methods section.*
☒	☐	A description of all covariates tested
☐	☒	A description of any assumptions or corrections, such as tests of normality and adjustment for multiple comparisons
☐	☒	A full description of the statistical parameters including central tendency (e.g. means) or other basic estimates (e.g. regression coefficient) AND variation (e.g. standard deviation) or associated estimates of uncertainty (e.g. confidence intervals)
☐	☒	For null hypothesis testing, the test statistic (e.g. *F*, *t*, *r*) with confidence intervals, effect sizes, degrees of freedom and *P* value noted<br>*Give P values as exact values whenever suitable.*
☒	☐	For Bayesian analysis, information on the choice of priors and Markov chain Monte Carlo settings
☒	☐	For hierarchical and complex designs, identification of the appropriate level for tests and full reporting of outcomes
☐	☒	Estimates of effect sizes (e.g. Cohen's *d*, Pearson's *r*), indicating how they were calculated

Our web collection on statistics for biologists contains articles on many of the points above.

Software and code

Policy information about availability of computer code

Data collection	NDP.view2; ImageJ 1.53e with plugin of “IHC Profiler” ; BD FACSDiva 9.0.1; FV10-ASW 3.1
Data analysis	FlowJo V10, GraphPad Prism 8, Microsoft Excel (version 2019), CellProfiler (v4.2.1), Cell Ranger (version 7.0.0) , Partek Flow (version 10.0.23.0214). Specifically, the "Split by feature type", "PCA", "Graph-based clusters", "UMAP" tools from Partek Flow were used for the scRNAseq analysis, with the parameters specified in relevant sections in the Methods.

For manuscripts utilizing custom algorithms or software that are central to the research but not yet described in published literature, software must be made available to editors and reviewers. We strongly encourage code deposition in a community repository (e.g. GitHub). See the Nature Portfolio guidelines for submitting code & software for further information.

Data

Policy information about availability of data

All manuscripts must include a data availability statement. This statement should provide the following information, where applicable:

- Accession codes, unique identifiers, or web links for publicly available datasets
- A description of any restrictions on data availability
- For clinical datasets or third party data, please ensure that the statement adheres to our policy

scRNA-Seq data that support the findings of this study have been deposited in the Gene Expression Omnibus (GEO) under accession codes GSE231322. The single-cell RNA sequencing datasets of human HCC analyzed in this study include those published by Zheng et al.34 and Zhang et al.32. The accession code of Zheng et al.

is GSE98638; The accession codes of Zhang et al. are GSE140228 and EGAS00001003449.
The human liver hepatocellular carcinoma (TCGA-LIHC) data were derived from the TCGA Research Network: http://cancergenome.nih.gov/.
Source data for Extended Data Fig. 10b-d have been provided as Source Data files. All other data supporting the findings of this study are available from the corresponding author on reasonable request.

Research involving human participants, their data, or biological material

Policy information about studies with human participants or human data. See also policy information about sex, gender (identity/presentation), and sexual orientation and race, ethnicity and racism.

Reporting on sex and gender	N/A
Reporting on race, ethnicity, or other socially relevant groupings	N/A
Population characteristics	N/A
Recruitment	N/A
Ethics oversight	N/A

Note that full information on the approval of the study protocol must also be provided in the manuscript.

Field-specific reporting

Please select the one below that is the best fit for your research. If you are not sure, read the appropriate sections before making your selection.

☒ Life sciences ☐ Behavioural & social sciences ☐ Ecological, evolutionary & environmental sciences

For a reference copy of the document with all sections, see nature.com/documents/nr-reporting-summary-flat.pdf

Life sciences study design

All studies must disclose on these points even when the disclosure is negative.

Sample size	Pilot experiments were used to estimate the sample size necessary to generate statistically significant results using the appropriate statistical tests. Genetically modified mice and their littermate controls were used for all experiments where possible. For comparing liver weights and nodule numbers, 5-10 mice/group was sufficient to achieve statistical significance. For survival curves, a cohort of 10-20 mice per group was used. To account for potential technical failures, including missed hydrodynamic injection and early mortality associated with injection, we usually included an extra 10% of mice/group. Early mortalities (<5 days) were considered to be due to injection-associated death and removed from the analysis. The exact numbers (n values) used in the study are indicated in the Figure Legends.
Data exclusions	Early mortalities (< 5 days) were considered to be due to injection-associated death and removed from the analysis.
Replication	The numbers of replicates and independent experiments have been stated in the Figure Legends. The attempts at replication were successful.
Randomization	To generate statistically appropriate numbers, it was usually necessary to use more than 3 litters of mice for each experiment. To control for the treatments (including plasmids and antibodies), mice from each litter were randomly divided into groups so as to guarantee that gender-matched and genotype-matched individuals obtained different treatments. This grouping was performed ahead of each experiment.
Blinding	For vector delivery by hydrodynamic injection, blinding was achieved during injection by placing littermates of different genotypes into new cages lacking mouse information. Blinding was also performed for quantitative analyses of liver sections.

Reporting for specific materials, systems and methods

We require information from authors about some types of materials, experimental systems and methods used in many studies. Here, indicate whether each material, system or method listed is relevant to your study. If you are not sure if a list item applies to your research, read the appropriate section before selecting a response.

Materials & experimental systems

n/a	Involved in the study
☐	☒ Antibodies
☒	☐ Eukaryotic cell lines
☒	☐ Palaeontology and archaeology
☐	☒ Animals and other organisms
☒	☐ Clinical data
☒	☐ Dual use research of concern
☒	☐ Plants

Methods

n/a	Involved in the study
☒	☐ ChIP-seq
☐	☒ Flow cytometry
☒	☐ MRI-based neuroimaging

Antibodies

Antibodies used

Antibodies used for flow cytometry included anti-mouse CD4 BUV737 (612843), anti-mouse CD8 PerCP-Cy5.5 (551162), anti-mouse CD19 BUV395 (563557), anti-mouse CD25 AF647 (clone 7D4 , 563598) and anti-mouse CD62L BUV737 (612833) from BD; anti-mouse CD45.2 Alexa Fluor 700 (109822), anti-mouse NK1.1 BV605 (108740), anti-mouse CD11b BV510 (101263), anti-mouse CD44 Alexa Fluor 700 (103026), anti-mouse OX40 PE (119409), anti-mouse CD4 BV510 (100559), anti-mouse CD45 PerCP-Cy5.5 (103132), TCRvβ5.1/5.2 PE-Cy7 (139508), anti-mouse CD25 (clone PC61 , 102016) PE-Cy7, anti-mouse CD4 PE (100408), anti-mouse CD62L FITC (104406), anti-mouse CD44 APC (103012), anti-mouse CD8 APC-Cy7 (100714), anti-mouse PD-1 APC (109112), anti-mouse Tim-3 PE (119704), anti-mouse Lag-3 PerCP-Cy5.5 (125219), anti-mouse PD-L1 PE-Cy7 (124314), anti-mouse CTLA-4 PE (106306), anti-mouse IFN-γ APC (505810), and anti-mouse IL-17A BV605 (506927) from BioLegend; and anti-mouse FOXP3 PE (clone FJK-16S, 12-5773-82) from Thermo Fisher. These antibodies were used at a 1:200 dilution. Mouse CD1d PBS-57 BV421-labeled tetramer was from the NIH Tetramer Facility. We determined the appropriate concentration of the CD1d tetramer by conducting a pilot experiment on each lot, and either a 1:1000 or 1:400 dilution was used.
Anti-GFP Alexa Fluor 488 (Thermo Fisher, A21311) were used to label GFP in intracellular staining analyses (1: 200 dilution).
Primary antibodies used for IHC included goat anti-GFP (Novus, NB100-1678, 1:750 dilution), rat anti-Foxp3 (Thermo Fisher, 14-5773-82, clone FJK-16s, 1:500 dilution), rabbit anti-CD3 (Abcam, ab5690, 1:400 dilution), rabbit anti-CD11b (Abcam, ab133357, 1:2000 dilution), rabbit anti-p53 (Vector Labs, VP-P956, 1:750 dilution), rabbit anti-c-Myc (Cell Signaling, #5605, 1:400 dilution), and rabbit anti-Pten (Cell Signaling, #9559, 1:200 dilution). Polymer-conjugated secondary antibodies included AP goat anti-rat IgG (MP-544415), HRP goat anti-rat IgG (MP-5444), HRP horse anti-goat IgG (MP-7405), HRP horse anti-rabbit IgG (MP-7405), and AP horse anti-rabbit IgG (MP-5401) (all from Vector Laboratories). The secondary antibodies are all applied directly from stock dropper bottle without further dilution.
Antibodies used to deplete mice of CD8+ T cells (anti-CD8, clone 2.43), CD25+ Tregs (anti-CD25, clone PC-61.5.3), NK cells, (anti-NK1.1, clone PK136), or CD4+ T cells (anti-CD4, clone GK1.5), or for blockade of PD-1 (anti-PD-1, clone RMP1-14), and isotype control antibodies were from BioXCell. To activate TCR signaling, hamster anti-CD3 (Biolegend, 100359, clone 145-2C11) and rabbit anti-Hamster (Jackson ImmunoResearch, 307-005-003) antibody were used.

Validation

The validation of all primary antibodies for the species and application is available from manufacturers (validation statements on the manufacturer’s website).

Animals and other research organisms

Policy information about studies involving animals; ARRIVE guidelines recommended for reporting animal research, and Sex and Gender in Research

Laboratory animals

Chat-GFP (B6.Cg-Tg(RP23-268L19-EGFP)2Mik/J), Chat-flox (B6.129-Chattm1Jrs/J), CD4-Cre (Tg(Cd4-cre)1Cwi/BfluJ), Il21r-/- (B6.129-Il21rtm1Kopf/J), OT-II (B6.Cg-Tg(TcraTcrb)425Cbn/J), Confetti (Gt(ROSA)26Sortm1(CAG-Brainbow2.1)Cle/J), Foxp3YFP/Cre (B6.129(Cg)-Foxp3tm4(YFP/icre)Ayr/J), NSG (NOD.Cg-Prkdcscid Il2rgtm1Wjl/SzJ) and control NOD/ShiLtJ mice were all purchased from the Jackson Laboratory and bred in the animal facility at the Princess Margaret Cancer Centre. The mice were housed on ventilated racks supplied with autoclaved microisolator cages. Reverse-osmosis (RO) water was supplied through an automatic watering system. The light cycle was lights-off at 6pm and lights-on at 6am. The ambient temperature was held between 22-23°C with humidity of 40-60%. Mice were routinely fed on the irradiated 7012 Teklad LM-485 Mouse/Rat Sterilizable Diet.

Wild animals

No wild animals were used in this study

Reporting on sex

Both the female and male littermates were used in this study. In most cases, multiple litters, including both sexes, were pooled to reach a predetermined sample size. In vitro assays utilized cells from sex-matched littermates of various genotypes, and alternated males and females in replicate experiments.
The single-cell transcriptomic analysis was performed on two males and two females for the control group and another two males and two females from their littermates for the HCC group. Single cells from individual mice were hash-tagged to enable the retrieval of sex-specific information, which was displayed in the figures (Fig. 4a,b, and Extended Data Fig. 4).
The exception is that only male mice were used for the NASH-induced liver cancer model. Based on our previous experience, the overall NASH syndrome and HCC incidence in females are much lower than in males. We chose to use males to efficiently evaluate the effects of Chat-expressing T cells in the HCC incidence in this model.

Field-collected samples

This study did not involve field-collected samples

Ethics oversight

All animal experiments were approved by the University Health Network Animal Care Committee.

Note that full information on the approval of the study protocol must also be provided in the manuscript.

Flow Cytometry

Plots

Confirm that:

☒ The axis labels state the marker and fluorochrome used (e.g. CD4-FITC).

☒ The axis scales are clearly visible. Include numbers along axes only for bottom left plot of group (a 'group' is an analysis of identical markers).

☒ All plots are contour plots with outliers or pseudocolor plots.

☒ A numerical value for number of cells or percentage (with statistics) is provided.

Methodology

Sample preparation	Liver tissues were collected, disrupted, and passed through 70-µm sieves to obtain single-cell suspensions. Mononuclear cells (MNCs) were enriched by centrifugation through a 40/80% Percoll gradient for 20 min at 2000 rpm.
Instrument	BD LSRFortessa™ cell analyzer, BD FACSAria™ Fusion cell sorter
Software	BD FACSDiva 9.0.1; FlowJo V10
Cell population abundance	For the Tconv and Treg sorted from Foxp3-YFP/Cre mice, or Chat-GFP+CD4+ and Chat-GFP-CD4+ T cells sorted from the HCC-bearing livers and control livers, the purity was above 90% by post-sorting analysis with flow cytometry.
Gating strategy	The preliminary FSC/SSC gates were adapted to include all the viable leukocytes (DAPI-CD45+). For the distinct cell populations such as CD4+ T cells, CD8+ T cells, B cells and et al., the specific markers are strong enough to separate the from other cell populations. To determine the positive and negative staining of other cells, such as the GFP positive, cytokine positive, and transcription factor positive, WT controls or FMO controls were included to determine the boundaries.

☒ Tick this box to confirm that a figure exemplifying the gating strategy is provided in the Supplementary Information.
